# The systemic and governmental agendas in presidential attention to climate change in Mexico 1994–2018

**DOI:** 10.1038/s41467-019-14048-7

**Published:** 2020-01-23

**Authors:** Arturo Balderas Torres, Priscila Lazaro Vargas, Jouni Paavola

**Affiliations:** 1 Centre for Climate Change Economics and Policy (CCCEP), School of Earth and Environment, University of Leeds, Leeds, LS2 9JT UK; 2 Centro de Investigación y Proyectos en Ambiente y Desarrollo (CIPAD), CP 45050 Zapopan, Mexico; 3 Environmental Engineering, Instituto Tecnológico y de Estudios Superiores de Occidente (ITESO), CP 45604 Tlaquepaque, Mexico

**Keywords:** Climate-change policy, Government

## Abstract

Ambitious climate action requires sustained long-term attention from political leaders. To understand how climate change entered the political agenda in a developing country, we examine from an agenda-setting perspective the attention paid by Mexican presidents to this issue from 1994 to 2018. We perform a longitudinal analysis of 968 documents referring to climate change published by four presidencies to describe changes in attention levels over time and to determine how changes in international agreements and public policies (i.e. systemic agenda) and National Development Plans (NDPs)(i.e. governmental agenda) influence them. Our results indicate international agreements and national legislation establish a baseline for inclusion of climate change into governmental actions. Agenda changes driven by international agreements result in reactive changes in attention, while ambitious approaches are aligned with proactive NDPs. Our results also indicate public awareness and electoral periods can open windows of opportunity for reframing agendas and promoting ambitious climate action.

## Abstract (web summary)

It is important to gain a better understanding on the contributing factors fostering climate action in developing countries. Here, the authors investigate the attention levels paid to this issue in the planning and implementation stages of climate policies in Mexico during 1994-2018, and find that international negotiations and executive governmental plans are strong drivers of the climate policy discourse in Mexico and likely to be so for developing countries more generally.

## Introduction

Ambitious climate action requires that policy and decision-makers focus on the issue in the long term^1^. Agenda-setting studies seek to elucidate why some problems emerge and are included in the public agenda and policies (e.g. in refs. ^1,2^). Since individuals and institutions have a limited capacity for attention (e.g. in refs. ^1,3–6^), understanding how problems capture the leaders’ attention is critical^6^ as the level of attention indicates the importance of an issue and has agenda-setting effects^4,7,8^. Our objective is to investigate from an agenda-setting viewpoint how the attention to climate change paid by Mexican presidents evolved from 1994 to 2018: in particular, we assess the effect of changes in the governmental and systemic or legal agendas on the level of attention.

Interest in environmental issues, such as climate change rise and fall over time^1,2,9–11^, but it is in periods of high interest when new institutions and policies to address them are adopted^9,12^. Policies and attention cycles can have long stable periods alongside short and intense periods of agenda and policy change, which can have long-lasting effects (i.e. punctutated equilibrium^10^). In his classic work Kingdon^2,13^ proposed that three conditions need to be met for an issue to enter an agenda: a problem is recognized, solutions are available and political conditions are favorable. A window of opportunity arises when the three conditions are met and policy change can be promoted^13^. The complementary punctuated equilibrium model suggests that changes in interests and policy processes are linked to changes in the definition of issues and relevant institutions^6^.

Earlier research has shown that attention to climate change is affected by new scientific information, feedback on implemented actions and focusing events^1,4,14^. Historically, developing countries had little interest in climate policy because economic development was seen to require industrialization and increased carbon emissions^15^. Climate policy was thus not a priority for them and any interest in it was at first based on international consensus and only later on domestic support^16^. Following the negotiations under the United Nations Framework Convention on Climate Change (UNFCCC) climate policy has spread from international to national and sub-national levels^17,18^. The attention paid to climate change is increasing both in international and national agendas^19^. In developing countries, drivers of climate policy include international negotiations^20^; potential access to international finance^21^; reputation^22^; interest in international decision making^20^; concerns about energy security and other local problems or co-benefits^15,23^; the sense of responsibility^24^; and interest to divert external pressure for more ambitious action^25^.

Earlier research often focused on advances in international negotiations^20,26^ and its focus on developed countries has left processes occurring in the developing world underresearched. The failure to reach an agreement in Copenhagen in 2009^27^ highlighted the role of emerging economies in climate change governance. Thereafter negotiations focused on inclusive long-term climate action which culminated six years later in Paris^28^. But pledges made under the Paris Agreement (PA) remain insufficient to prevent dangerous climate change^29,30^. Therefore, it is important to understand how climate change can gain importance in the policy agendas in Non-Annex I developing countries, which now account for 50% of annual greenhouse gas emissions globally^31^.

We contribute to addressing this evidence gap by examining the attention given to climate change by Mexican presidents over a 24-year period of four federal administrations during which climate change became a policy issue globally and nationally in Mexico (1994–2018). The administrations are those of Ernesto Zedillo Ponce de León (EZPL), Vicente Fox Quesada (VFQ), Felipe Calderón Hinojosa (FCH), and Enrique Peña Nieto (EPN). Our analysis covers three administrative changes and two changes in the political party in power. EZPL and EPN represented the Institutional Revolutionary Party (PRI), a centrist pragmatic party. VFQ and FCH are members of the National Action Party (PAN), a party close to conservative investors and private sector. Common to the four administrations is that they all implemented neoliberal economic policies (e.g. in ref. ^32^) and that since 1997, their political parties did not have majority in the federal Congress (e.g. in ref. ^33^). In presidential regimes such as that of Mexico, the leadership of the executive is critical in setting the priorities for public policies^6^.

Following agenda-setting scholars, we consider that the political agenda consists of: first, the public agenda or the issues of interest to the general public; second, the systemic agenda of issues addressed by legislation, and; third, the governmental agenda of issues proactively and explicitly considered in public decision making^1,34,35^. We perform a longitudinal study using content analysis^36^ to build a chronology of attention. Our evidence base is the documents referring to climate change and published by the presidential offices over the 24-year period (*n* = 968). We study how changes in the governmental and systemic agendas^34,35^ relate to the changes in and patterns of presidential attention to climate change in Mexico.

For our purposes, we define the systemic agenda as the policies and activities originating to international agreements and enacted domestic climate legislation, while the governmental agenda is defined in National Development Plans (NDPs)^37–40^. In Mexico, signed international agreements have a high legal status only just below the Constitution^41,42^. The NDPs in turn are mandatory legal instruments which guide public action and establish the priorities, objectives and programs of the administration^43^. Development planning is not exclusive to Mexico: it has been common in many developing countries^44–47^ since the 1960s. Therefore, improved understanding of how NDPs as a key aspect of governmental agenda relate to climate policy and attention cycles has wider relevance.

Few studies have analysed to date how NDPs address environmental issues (e.g. in refs. ^23,48^). We contribute new quantitative evidence from the analysis of a large data set on attention patterns, which is particularly novel in research on climate policy in non-Annex I countries.

In what follows, we describe key developments in climate policy in Mexico before presenting the results of our analysis. Our findings indicate that changes in the governmental and international systemic agendas resulted in discernible changes in attention levels paid by Mexican presidents to climate change. We find that international agreements and domestic legislation provide a bottom line for addressing climate change, but that ambitious climate action is based on ambitious executive governmental plans.

## Results

### Background on climate policy in Mexico

Mexico provides a good case to understand climate action^49^. Despite being a middle-income, non-Annex I country contributing <2% of the global GHG emissions^50^, it was one of the first countries to adopt a voluntary mitigation target in 2008 and national climate legislation in 2012^51–53^. To understand the evolution of Mexican climate action it is important to acknowledge that as a non-Annex I country to the UNFCCC it did not adopt a legally binding mitigation target under the Kyoto Protocol (KP). The Annex I was based on membership of the Organisation for Economic Cooperation and Develompent (OECD) at the time^20^. The text of the UNFCCC was prepared in 1992 at the Rio Summit and ratified later in March 1994; Mexico joined the OECD only a few months later in May 1994. The country collaborated closely with the OECD since the early 1980s and the process to join it accelerated in 1993^54^. By the time Mexico joined the OECD, it was the ninth largest economy in the group; yet on per capita income basis Mexico was at the bottom of the group just above Turkey^55,56^. This increased expectations of and pressure to adopt commitments on climate action^57^ and to demonstrate leadership in it^58^.

Before 1992, climate change discussions in Mexico were limited to the Ministries of Foreign Affairs (MoFA) and Environment (MoE)^59^. Academics from Universidad Nacional Autónoma de México (UNAM, the acronym in Spanish) became involved in research and debates^58^ and their supportive approach on climate negotiations informed and influenced Mexico’s involvement over longer term^58^. Domestic relevance of climate change increased after the entry into force of the Convention and when the negotiations on the KP started in 1995^57^. At the end of the 1994–2000 administration, Mexico ratified the KP and developed a National Strategy on Climate Change (NSCC) which, however, was not implemented^58^. The withdrawal of the U.S. from the KP reduced the prospects for a carbon market in North America at the beginning of the 2000–2006 administration, thus weakening interest in the topic^58^. But after the European Union (EU) ratified KP, interest in climate policy and the KP’s Clean Development Mechanism (CDM) increased again^58,59^. This administration also published a NSCC, but it was not implemented as this happened in the last months of its 6-year term^49^.

In the 2006–2012 term, climate action included the early publication of a third NSCC^60^ in 2007 and the first Special Program on Climate Change (SPCC)^61^ in 2009, the adoption of a voluntary mitigation target, a proposal for the Green Climate Fund (GCF), the organisation of the COP 16 in Mexico and the enactment of the General Law on Climate Change (GLCC) at the end of the administration^49,53,62^. Initiatives to reduce emissions from deforestation and forest degradation (REDD+) (e.g. in ref. ^63^) and reforestation programs were also developed (e.g. in ref. ^64^).

In the most recent 2012–2018 term, climate action continued under the framework of the GLCC but it had lower priority and limited funding^65^. The term witnessed the update of the NSCC^66^ and the SPCC^67^; the creation of a carbon tax^68^; the publication of the Nationally Determined Contribution (NDC); and the development of energy projects. The national REDD+ strategy was published in August 2017 and the design and creation of a cap-and-trade system for GHGs advanced in the final months of 2018^69,70^. The GLCC was reformed in May 2018^71^ to formally include the pledges of the NDC^65^.

### General assessment of climate action

Mexico’s climate leadership^59^ consists mostly of pledges and adoption of legislation but its actions have not yet changed its greenhouse gas emissions^49^. Nevertheless, it is possible to characterize the performance of early actions using published and official information. Between 1994 and 2006 there were few advances yielding measurable results as the institutional frameworks were not yet in place. It is notable that the first two administrations published their NSCC at the end of their terms, indicating the issue had a low priority. The KP marked a turning point for climate action. It was signed in 1997 but only after 2005 was it possible to start its implementation. The number of CDM projects provides one proxy of climate action at this stage. A total of 192 Mexican projects are registered under the CDM and they are expected to mitigate 283.9 MtCO_2_e by 2030^72^; a significant 42% of 2015 emissions^73^. A third of the projects were registered under the VFQ’s administration, 52% under FCH’s and 15% under that of EPN’s. VFQ’s administration clearly took up the opportunities created by the KP and CDM projects thrived under FCH. EPN’s administration underperformed with regard to CDM although Mexico signed the Doha Amendment^74^ and created in 2014 a flexible carbon tax aiming at stimulating domestic demand for CDM certified emission reductions^68^, which however was not implemented^65^.

The implementation of the SPCCs exemplifies action in the most recent 2006-2018 period. The 2009–2012’s SPCC^61^ had an objective to reduce 51 MtCO_2_e/year and by 2012 the results exceeded its goal by 4%^75^. The 2014–2018’s SPCC in turn set an objective to reduce 83.2 MtCO_2_e/year^67^ but by 2018 only achieved 37.2% of the target^76^. Although the rate of increase of emissions diminished in 2010–2015, the yearly increase is still of about 0.9% per year^73^. The energy sector was an important priority for EPN’s administration, but the emission factor of the national electricity grid increased 16% from 2014 to 2018^77,78^ evidencing the lower priority given to renewable energy and climate change in the energy sector strategy.

Climate action can also be explored considering resources dedicated to it, particularly through the environmental sector. Budget allocated to the MoE increased under FCH’s term: the last budget allocated in 2012^79^ by his administration was 212% higher than that allocated in 2006^80^ in the final year of VFQ (converted to U.S. dollars)^81^. When adjusted by inflation in the same period^82^ the increase was 165%. The budget of the MoE in 2018^83^ at the end of EPN’s period was only 47% of that granted in 2012^79^ (37% when adjusted with inflation^82^). Mexico was the world’s second largest recipient of multilateral climate finance between 2004 and 2014 after Morocco^84^ but has since fallen to the fourth place^85^.

### A chronology of presidential attention

**Fig. 1:**
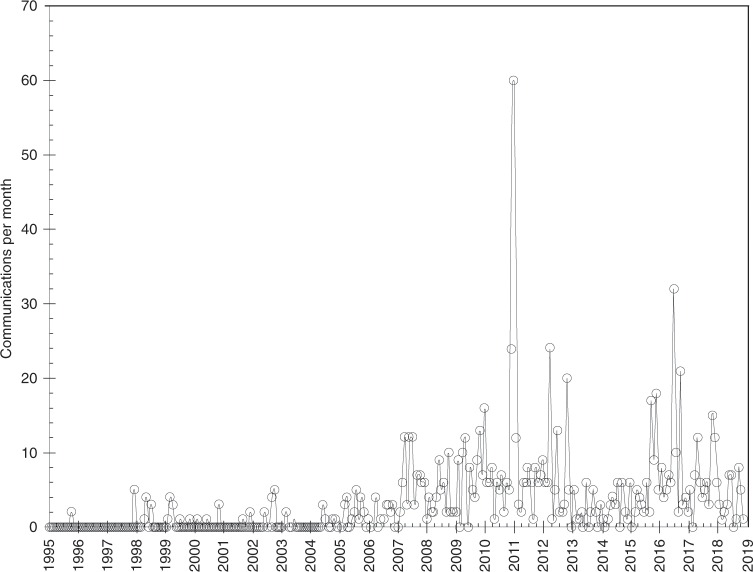
Number of publications mentioning climate change per month published in the public websites of presidential offices in Mexico. The period spans four federal administrations from December 1994 to November 2018; points represent the total number of communications published by presidential offices mentioning climate change retrieved from the public websites per calendar month.

A total of 968 communications published by presidential offices referred to climate change in the period of analysis (30 by EZPL, 65 by VFQ, 517 by FCH, and 356 by EPN); the list is presented in the supplementary online material (Supplementary Data 1). Figure 1 indicates the number of documents referring to climate change by month.

Figure 1 shows that attention paid by Mexican presidential administrations to climate change exhibits similar patterns of ups and downs reported in previous studies from elsewhere (e.g. in refs. ^4,14,86^). Before 1998 there were practically no references to climate change and presidential mentions start in late 1997 to the processes around the KP. This corroborates that while climate change was discussed at the time domestically in a bottom-up way^58^ and in the context of international negotiations, it had not reached the attention of the executive. Mentions became more frequent in 2005 and specifically after 2007, reaching the maximum in late 2010 driven by the organisation of COP 16 in Cancun. From 2013 to mid 2015 attention declined, to increase again in late 2015 driven by the celebration of COP 21 in Paris and the participation of EPN in North American fora in summer 2016^87,88^. Attention then declined in 2018. In the next sections, we will outline the main changes in the systemic and governmental agendas during this period to help understand these changes in attention.

### International agreements and domestic legislation

The inclusion of climate change into the systemic agenda has been gradual. The signing of the KP in 1997 and the Kyoto Protocol’s entry into force (KPEIF) in 2005, were the first important developments in the period of analysis. They enabled the development of CDM projects and untapped international climate finance. The next milestones were the enactment of the GLCC in 2012, the adoption of the PA in 2015 and the reform of the GLCC in 2018. The organisation of COP 16 in 2010 was important for presidential attention. COPs are not only international focusing events, but a period during which the systemic agenda is created, reviewed and revised. The systemic agenda based on agreements under the UNFCCC did not translate into responsibilities, strategies and policies for practical implementation until domestic legislation was adopted. Before this happened, advances were contingent to direct executive mandates and mostly relied on external development of institutional frameworks under the UNFCCC.

After COP 16, Mexico’s role in the UNFCCC process was deemed so successful for restoring the negotiation process, that a few years later in 2016 Patricia Espinosa, who was Minister of the MoFA, became the Executive Secretary of the UNFCCC (e.g. in refs. ^89^). Actions developed in the 2006–2012 term were innovative and ambitious, however they had no legal protection from political changes until the GLCC was enacted^65^. The law included targets conditional to the adoption of an international agreement and provision of adequate international finance, to reduce emissions 30% by 2020 and 50% by 2050^52,53^. The fact that these goals were aspirational and involved 2020 and 2050 as the key points in time for evaluation undermined accountability as there were no targets for all administrations spanning the timeframe (e.g. the 2012–2018). The law has been crucial in keeping climate change on the governmental agenda but it lacks concrete mechanisms for implementation, particularly regarding financing, coordination of climate action and its monitoring and evaluation^65^. Some challenges of the implementation of the GLCC relate to its design, while others relate to difficulties in implementation processes and obstacles related to financial capacity and political will^65^. Three years later, when the PA was adopted in 2015, Mexico formalized its pledges for climate action at the international level. The goals of the GLCC were updated and communicated by defining conditional and non-conditional targets for 2030^90^. These new targets defined in the NDC were incorporated into the GLCC in May 2018^71^.

### National development plans

Next we examine how climate change was defined in the governmental agendas. Table 1 below reports the number of references to climate change and how it was described and addressed in the NDPs.

**Table 1:** Mentions and definition of climate change in Mexican NDPs (1994–2018).

Term	Mentions	Description
1994–2000, EZPL	0	Climate change is not mentioned or considered a problem. However, it refers to problems associated with causes or consequences of climate change but without mentioning it (e.g. deforestation, morbidity, urban air pollution, migration, solid waste and waste water management). Total number of words 70,494; mentions of climate change per 10,000 words: 0.00.
2000–2006, FVQ	5	Climate change is mentioned as a polemical issue and the level of development in Mexico is considered to prevent the adoption of emission reduction targets. Mitigation measures would be promoted in the energy sector if they do not threaten national development. Need for adaptation and impacts of climate change are not mentioned. Total number of words 88,206; mentions of climate change per 10,000 words: 0.57.
2006–2012, FCH	39	Climate change is acknowledged as an unequivocal environmental problem. It is characterized with statistics and scientific information; the NDP has specific sections for mitigation and adaptation and specific objectives, strategies and actions. Sources of emissions, sectorial actions and relevant actors are identified. There is willingness to play an active and relevant role in international negotiations and the need to increase the global scale of mitigation efforts is recognized. Total number of words 105,202; mentions of climate change per 10,000 words: 3.71.
2012–2018, EPN	18	The NDP mentions climate change in the context of natural risks without elaborating on the causes or options for mitigation and adaptation. There are no specific sections or objectives for climate action. The link between economic development and carbon emissions is acknowledged but climate action is only described at the level of strategies and actions and specific objectives are not set; it is subordinated to more general objectives of green growth and transition to a low carbon economy thus not elaborating on adaptation. Previous leadership at international level is acknowledged. The country will maintain a role of a committed participant in international processes to defend national interests. Total number of words 68,728; mentions of climate change per 10,000 words: 2.62.

Climate change was not even mentioned in the NDP between 1994 and 2000. However, the plan mentioned concerns and actions that relate to aspects of climate mitigation and adaptation. Thus some actions and policies moved forward, but without a strategic approach. This could be expected given that concrete institutional frameworks for climate action had not yet been developed, domestically or globally. The situation did not change much in the next administration which described climate change as a polemical issue whilst leaving the window open for no-regret opportunities. This explains partly how the KPEIF reinvigorated domestic interest in the issue^59^. The initial reluctance to reduce emissions is consistent with the non-Annex I concern at the time that climate action might adversely affects economic development, oil production^20,58^, and in the case of Mexico, public budget.

Climate change was significantly redefined in the 2007–2012’s NDP. It included the highest number of references to the issue and presented a comprehensive description of the problem, implications and possible solutions and their translation into proactive implementation. Reference to the need for greater global mitigation ambition in the plan suggests why Mexico adopted a voluntary goal in 2008. The aspiration for a proactive international role also offers insight into the origins of the Mexican climate fund proposal and interest to organize COP 16.

The governmental change in 2012 brought another redefinition of climate change as a public problem. It was now mentioned in the context of natural disasters and international negotiations but not discussed in terms of its causes; specific mitigation and adaptation objectives were not defined. Climate change was deemphasized and subordinated to strategies promoting green growth and low carbon development. However, the institutional framework already in place (i.e. GLCC) helped to keep climate change in the executive agenda and other areas of the government.

### Agenda changes and changes in attention levels

**Fig. 2:**
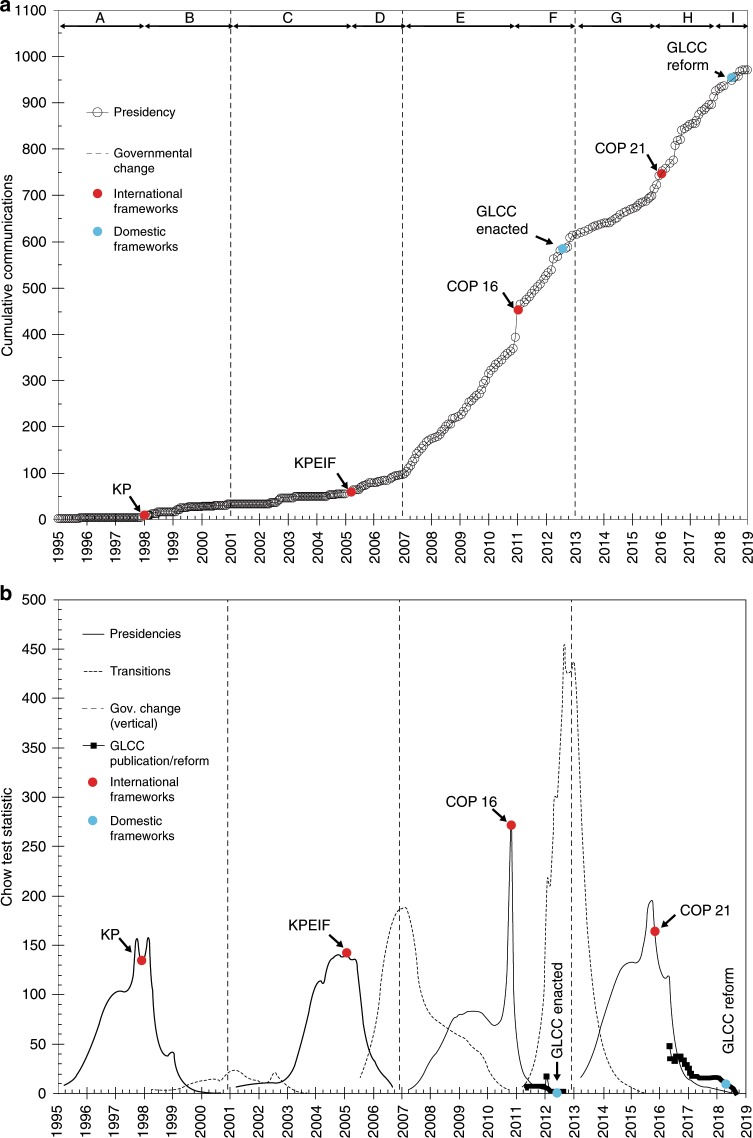
Evolution of attention to climate change by Mexican presidents from 1994 to 2018 as depicted by the trends in the cumulative number of communications. Governmental changes and the dates of key developments in international and domestic frameworks are identified. **a** Presents the cumulative monthly communications and key events modifying the systemic and governmental agendas; the lower **b** Presents the Chow test statistic to test the structural differences in attention levels.

We plot the cumulative number of monthly mentions of climate change in Fig. 2 to examine the change in the trend and not the discrete monthly variations (Fig. 1). The smoother pattern enables the identification of periods with more or less stable attention levels (i.e. the period from 1998 to 2005 is particularly stable). Figure 2 also shows the dates of changes of administrations and the months when the main developments in the systemic agenda occured.

The level of attention in terms of the slope changes around the dates of the relevant international events and around 2006/2007 and 2012/2013. The lower panel in Fig. 2 presents the Chow test statistics^91^ obtained when analyzing the structural changes for the segments of interest. Results show that for each of the four presidencies, the Chow test statistic peaks around the dates of the selected international events. This confirms that these events produced important structural changes in presidential attention to climate change. However, this is not the case for the enactment and reform of the GLCC. These events did not produced a structural change in presidential attention. The impact of the GLCC enactment in June 2012 and its reform in May 2018 on presidential attention is challenging to assess. These events occurred only a few months before the end of the pertinent administrations leaving a short time for a reaction. Also, it is not possible to know what the level of attention of the following presidencies would have been had the legislation not been enacted. Finally, it can be seen that the attention patterns in the last months of the leaving administrations and the first months of entering ones in 2006 and 2012 are structurally different and data cannot be pooled together as the Chow test statistic presents prononunced peaks. The governmental change in December 2000 is more subtle indicating similar attention patterns before and after this date.

We identify nine segments with different slopes (Table 2) which also maximized the coefficients of determination (*R*^2^). In all cases the regressions are significant at 0.01; after the signing of the KP in all cases the *R*^2^ are higher than 90%.

**Table 2:** Regression analysis in the level of attention of Mexican presidents to climate change after changes in governmental and systemic agendas (1994–2018).

Identification-Code	EZPL-1	EZPL-2	VFQ-1	VFQ-2	FCH-1	FCH-2	EPN-1	EPN-2	EPN-3
Segment for Regression	A	B	C	D	E	F	G	H	I
Start date	Dec-94	Jan-98	Dec-00	Mar-05	Dec-06	Dec-10	Dec-12	Sep-15	Nov-17
End date	Dec-97	Nov-00	Feb-05	Nov-06	Nov-10	Nov-12	Aug-15	Oct-17	Nov-18
*n* (months)	37	35	51	21	48	24	33	26	13
Level of attention *b* (slope, comms/month)	0.101	0.625	0.611	1.634	5.878	7.003	2.552	7.772	3.698
Coefficient of Determination (*R*^2^)	51.3%	90.6%	93.4%	97.0%	98.9%	98.7%	98.4%	97.2%	97.8%

Comparison between segments	A and B	B and C	C and D	D and E	E and F	F and G	G and H	H and I	G and I
Change in Level of Attention, *b* (%)^a^	517%	−2.2%	167.5%	259.8%	19.1%	−63.6%	204.5%	−52.4%	44.9%
*t* value (absolute values)	13.52	0.33	14.63	38.08	5.75	24.30	18.87	12.80	6.45
Sig-Dif. Between Segments (*t*-test *α* = 0.01)	Yes	No	Yes	Yes	Yes	Yes	Yes	Yes	Yes

^a^Estimated as *b*_2_/*b*_1_ − 1

Presidential attention to climate change increased following changes in the international climate negotiations; this is reactive behavior. The dates when decisions were made at international events became inflection points. These increases were substantial for EZPL, VFQ, and EPN as attention increased by 517%, 167%, and 204%, respectively. In the case of FCH, the effect of COP 16 is smaller (i.e. 19%); the most visible effect is an upwards displacement of cumulative values in December 2010.

The level of attention at the beginning of EPN’s administration until August 2015 was lower than that of the previous government. During this period the focus of the executive was on structural reforms (e.g. energy, education, fiscal matters). The post-COP 21 period can be divided into two segments (H and I). Attention levels increased a couple of months before COP 21, to reach similar levels as those observed during in 2006–2012. During the last phase of this presidency (Segment I), the level of attention declined and as results of the evaluation of the SPCC 2014–2018 indicate^76^ attention did not translate into effective implementation.

Table 2 shows that following the 2000 elections there was no significant change in the slope despite the partisan change. Climate change was still not a priority and it was not yet defined as a salient public issue in the corresponding NDPs. In contrast, although the change in administration in 2006 did not involved a partisan change, the redefinition of climate change and its inclusion into the NDP indicates a significant difference in the attention level of this administration. These observations resonate with the punctuated equilibrium model^6^.

### Attention in the planning and implementation of the agenda

The governmental agenda is formally defined in the NDPs. To assess the level of attention to climate change during the planning of the governmental agenda in contrast to its implementation, we consider the communications retrieved from the presidential websites. To characterize the level of attention we plot the number of documents retrieved against the number and mentions in the NDP for each administration.

**Fig. 3:**
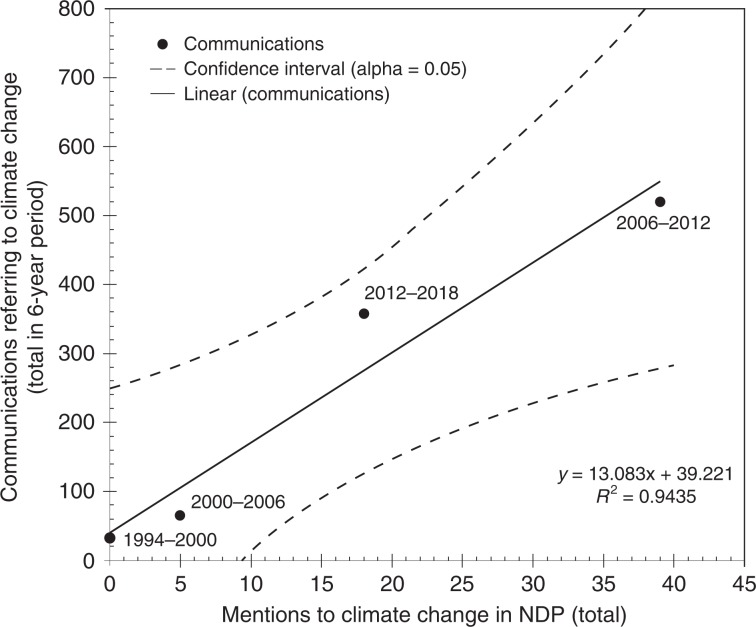
Relationship between the number of mentions in NDPs and the number of communications made by presidential offices referring to climate change for each presidency of Mexico (1994–2018). Data points indicate the total number of mentions and attention levels paid to climate change in the planning and the implementation of the governmental agendas in the corresponding 6-year periods; regression confidence interval for 95% level.

Figure 3 shows a consistent relationship between attention levels to climate change in the NDP during the planning of the governmental agendas and in the number of communications retrieved from the presidential websites during its implementation period. The coefficient of determination (*R*^2^) is 94% for a linear regression. This parameter is a good predictor of the level of attention observed during the past governmental periods. It is necessary to continue monitoring the public agenda and attention paid to different issues to generate a record for better understanding the processes associated with the planning and implementation of climate policy.

### Inclusion of climate change into the governmental agendas

Our results provide previously lacking evidence on how the NDPs critically construe the governmental agenda and are a good indicator of the expected attention levels in each governmental term. It is therefore worth looking at past changes in governmental agendas to explore how policy windows emerged and how Kingdon’s three streams confluenced at the time.

FCH’s term and NDP were a key for the inclusion of climate action into the Mexican policy agenda. Before running for presidency he was director of the public investment bank BANOBRAS and as Minister of Energy he was involved in renewable energy projects and procurement of international climate finance. Nevertheless, during the electoral campaign of 2006 climate change was not yet an issue and it was not part of his political platform^58^. In the two televised debates before the elections, he did not mention climate change and only once referred to the financial opportunities under KP when echoing a proposal of one of his opponents, Patricia Mercado^92,93^. Still, he made proposals and took positions that helped creating political conditions for the inclusion of the issue in the agenda. He promised in his discourses a responsible government with a proactive role in international negotiations and one open to the advice of scientists such as the Nobel Prize recipient Dr. Mario Molina who by that time had proposed a change in petrol formula sold in the country to improve air quality^92,93^. Climate change thus became included into the governmental agenda after these debates, between June 2006 and before the publication of FCH’s NDP in May 2007.

In 2006, Mexican newspapers gave limited attention to climate change and considered it a secondary issue^11^. This was also the case with newspapers in Spain^94^. Thus in the spanish-speaking world climate change was not in the public agenda. However, in newspapers published in english, attention to climate change increased substantially from September to November 2006 as a result of the publication of the Stern Review: The Economics of Climate Change^95^ and the release of Al Gore’s documentary An Inconvenient Truth^86^. These events helped construe climate change as an important problem with plausible solutions for the receptive FCH’s team preparing the NDP.

For the last administration considered, in the NDP and the first 2 years of EPN’s mandate less attention was given to climate change. It is impossible to identify all reasons behind a policy change^1,2,6^ but some can be hypothesized. The 2012 elections returned to power the new PRI (e.g. in ref. ^96^) and during the previous 12 years climate advocates might have not interacted with other political groups than PAN. Therefore, the agenda of the new government was framed around other issues: new administrations advance their own pet issues^1^. At this time, climate change was not an urgent problem. The country had already passed the GLCC in 2012, when solutions are already in place, incentives for attention are reduced as it can be perceived that the problem is solved^1,2^. The 2012/2013 policy window also coincided with a period of the lowest level of global public attention to climate change after 2007^97^; the attention only increased later in the runup to COP 21 in 2015. Nevertheless, the high sensitivity of EPN to public opinion at international level made him politically receptive to the issue and lead to higher reactive attention around COP 21. However, it faded away without becoming translated into mitigation efforts or higher budgetary priorities. Falling oil prices also reduced public revenue and climate action and the environmental agenda as a whole faced budget cuts^98,99^.

Mexico had a general election in July 2018 and Andrés Manuel López Obrador (AMLO) was elected after running for the presidency for the third time. This produced another partisan change to the leftist Movement for National Regeneration (MORENA). Two important changes followed. The new administration marks the end of centre-right neoliberal policies (e.g. in ref. ^32^) and the president obtained partisan majority in the federal Congress for the first time since 1997 (e.g. in ref. ^33^). A question remains whether presidential attention paid to climate change under these new circumstances will follow similar patterns as in the last four administrations (e.g. in Fig. 3). Climate change was not an important issue in AMLO’s 2006 and 2012 campaigns^58,65,100^. However, in the 2018 campaign his climate change platform was assessed by a group of environmental NGOs the best among the candidates^101^. Still, his campaign focused on poverty alleviation, vulnerable groups such as rural and indigenous communities, austerity and a fight against corruption. It is unclear how AMLO will advance the climate strategy proposed in the campaign. However, reframing the need for mitigation and adaptation actions around topics as the need to protect vulnerable groups in the context of poverty alleviation, or as means to reduce budget pressures via savings can help keeping the topic in the agenda (e.g. in ref. ^13^).

Since the end of 2018, global public interest on climate change has increased again, including in Europe and the U.S.^97^. This has been fuelled by the awarding of the Nobel Prize in Economics to William Nordhaus for his studies on the global economy and the climate, and the climate campaigns of swedish teenager Greta Thunberg and other social movements. If the momentum continues to build up and if the priority of climate change increases among the public, the governmental attention to the issue in Mexico might increase. Other foreseeable events that can alter presidential attention before 2024 in Mexico are the U.S. presidential elections in 2020 and the first revision of the implementation of the PA in 2023^28^.

## Discussion

We analyzed the NDPs and public information from 1994 to 2018 to characterize the evolution of presidential attention to climate change in Mexico. Our results are consistent with agenda-setting literature, particularly with Kingdon’s^2^ multiple streams model and the punctuated equilibrium model^6^ but also provide new empirical evidence that has been lacking to date. The consideration of the changes in the different agendas facilitates the understanding of the key observable developments of climate policy and action in Mexico in the last 24 years. We contribute to the existing body of knowledge by identifying the differential impacts of changes in the systemic and governmental agendas to leaders’ attention to climate change. Our results indicate that changes in the systemic agenda driven by international agreements under the UNFCCC resulted in reactive changes which are substantial but potentially reversible. In contrast, the comprehensive inclusion of climate change and responses to it into the governmental agenda lead to higher, more proactive and more stable attention levels. We can conclude that while international agreements and domestic legislation establish the minimum baseline for including climate change into governmental action^65^, proactive and ambitious action is underpinned by executive governmental plans.

A question remains whether governmental agendas of other countries evolve in a comparable manner than Mexico’s. This is an important area for future research given the prevalence of development planning globally (e.g. in refs. ^45,47^). It is also important to explore whether similar processes involving systemic and governmental agendas are at play at sub-national levels (e.g. State/Regional and Municipal/Local Level Development Plans) and among different implementing ministries and institutions (e.g. Ministry of Environment, Ministry of Energy, etc) to help further improving our understanding of the factors behind diffusion and mainstreaming of climate action.

As our results demonstrate, it is critical not only to keep track of the enactment of climate legislation^102,103^ and goals stated in the NDCs, but also to assess and promote the inclusion of climate action into governmental plans since they define the priorities and resources for practical implementation of such legislation and contributions. While we have focused here on levels of attention, it is important not to forget that, to fully evaluate policy implementation, the efficacy of climate actions needs to be assessed in terms of changes in emissions and levels of vulnerability, too.

## Methods

### General approach

We first performed a review of the literature on the main developments related to the design and implementation of climate action and associated policies in Mexico in the period of interest. This enabled us to identify the observable outcomes of implementation processes. It is within the context provided by these developments that we analyze whether attention levels match the actions implemented and if changes in the agendas of interest explain changes in attention levels and climate policy.

We performed a longitudinal study of attention paid by Mexican presidents to climate change from 1994 to 2018 using content analysis, a systematic approach to translate qualitative information into quantitative data using defined frameworks to analyze it, explore statistical relationships and to make inferences^36^. The method has been used to examine a range of issues including the attention given to climate change in newspapers in Canada^19^, Mexico^49^, and the U.S.^104^, and in scientists’ congressional testimonies on climate change in the US^105^. Coding of the content of information sources over time has been used to examine policy change in light of the punctuated equilibrium model, too^6^. In what follows, we explain how the material was retrieved, coded and analysed.

### Presidential attention on climate change

The Mexican federal government publishes different types of information on public websites as part of its communication and transparency policies, including transcripts of speeches, press releases, interviews, informative notes, and other official communications. The archieved websites of past Mexican presidencies remain online in the active presidential page^106–110^. We searched the four presidential websites for publications where the terms climate change, global warming or greenhouse effect occurred (e.g. in ref. ^104^). All documents found published by the presidential offices were retrieved and given an individual code number. A database was built including the title and date of publication. Our analysis focuses on these documents and not on strategic documents such as the National Communications submitted by the Mexican Government to the UNFCCC, or texts of specific legislation or laws since these documents are prepared by technical experts, by staff of technical bodies of the MoE or the Federal Congress, and not by the presidential offices. By focusing on the information published by the presidential office our objective is to generate a proxy of how level of attention to climate change in the closest circle of the execuctive has evolved over time.

### The systemic and govermental agendas

One of our objectives is to understand how the inclusion of climate change in the systemic and governmental agendas relates to presidential attention to climate change. In order to understand this, we describe when the main advances originating from the international agreements and domestic climate legislation occured and how they contributed to the definition of the systemic climate agenda in the country.

The governmental agenda is inscribed to the NDP which is published by each Mexican federal administration in the first months after coming into power in the country’s Official Gazzette, Diario Oficial de la Federación in Spanish (DOF). NDPs are prepared according to the Planning Law^43^. We accessed the DOF to obtain the NDPs of the four federal administrations from 1994 to 2018^37–40^. These were published on the 31 May 1995 by the administration of EZPL, 30 May 2001 by the administration of VFQ, 31 May 2007 by the government of FCH and 20 May 2013 by the administration of EPN. The four NDPs are substantial documents: their word counts vary between 67,000 and 105,000 words. To determine whether climate change is included in each governmental agenda we first made a search for the presence and frequency of mentions of climate change, global warming and greenhouse effect in the NDPs following the standard practice (e.g. in ref. ^104^), to obtain the total count of mentions of climate change. We then examined how climate change as a problem and potential solutions were framed. We could thus establish whether climate change was included in the governmental agenda of the four presidencies and whether it had been reframed or redefined in the NDPs in line with the punctuated equilibrium model. The dates of the governmental changes define temporal boundaries to verify if changes in the definition of the problem in the NDPs was associated to changes in attention levels during the presidential period.

### Analyses made

We first built a chronology of presidential attention to climate change considering the number of publications mentioning climate change per month as a metric of attention. Our hypotheses are that changes stemming from the systemic agenda might modify the presidential attention trends reactively, while changes in the governmental agenda might indicate a more proactive attention towards climate change. Considering that the executive has more control over the governmental agenda when climate change is explicitly included in the NDP in the planning stage, we hypothesize that it will also get more attention during the implementation phase; how a problem is defined provides information on how it will be dealt with^6,111^.

The dates of changes in administrations and of the key events shaping the systemic agenda are used to assess differences in the levels of attention and their patterns; the changes in administration represent the changes in the governmental agenda reflected in the corresponding NDPs. Based on the segmentation drawn from the consideration of these dates, we use the cumulative number of communications to identify periods with stable levels of attention and estimate the slopes and statistical differences of each of these periods through an ordinary least squares regression^91,112^. The Chow test statistic^91^ is used to identify structural breaks in attention patterns in the different periods identified; this is to confirm the dates around which data can be segmented and improve the resulting linear regressions. The Chow test statistic is obtained by comparing the information of three linear regressions: two presumably different groups of data and the pooled information of both groups.

For each administration, we estimated the linear regression of the cumulative communications over the whole period of 72 months and made 66 iterations and estimated the Chow test statistic for 66 possible divisions of the data in two segements between the 5th and 70th months; the maximum values of the Chow test statistic indicates the months at which the time series can be divided to analyze the data as different segments producing better results. The same approach is followed to assess structural breaks around the dates when governmental changes took place and when the GLCC was enacted and reformed. By comparing the differences in the slopes of the linear regressions we can test whether changes stemming from the governmental or systemic agendas alter the level of attention.

Finally, we compare the frequency of mentions of climate change in the NDPs with the number of documents retrieved from each presidential website to assess the similarity in attention levels between the governmental agenda at the planning phase at the start of each term and during each 6-year period when this is implemented. We test how well the data on attention levels at the planning and implementation phases fits to a linear regression using the number of mentions in NDPs as an independent variable; we compute the confidence interval for the regression at 95% level using a *t*-distribution.

## Supplementary information

Description of Additional Supplementary Files

Supplementary Data 1

## Data Availability

All data generated or analysed during this study are included in this published article (and its supplementary information files). The Supplementary Data 1 presents the database of the communications retreived from the presidential websites.

## References

[1] Pralle SB (2009). Agenda-setting and climate change. Environ. Politics.

[2] Kingdon, J. *Agendas, alternatives, and public policies* 2nd edn. (Longman, New York, 1995).

[3] Cobb R, Elder Ch (1984). Agenda building and the politics of aging. Policy Sci. J..

[4] Schafer MS, Ivanova A, Schmidt A (2014). What drives media attention for climate change? Explaining issue attention in Australian, German and Indian print media from 1996 to 2010. Int. Commun. Gaz..

[5] Hilgartner S, Bosk CL (1988). The rise and fall of social problems: a public arenas model. Am. J. Sociol..

[6] Baumgartner, F. R. & Jones, B. D. *Agendas and instability in American politics* 2nd edn. (University of Chicago Press, U.S, 2009).

[7] Dearing, J. W. & Rogers, E. M. *Agenda Setting*. (Sage, Thousand Oaks, 1996).

[8] Sampei Y, Aoyagi-Usui M (2009). Mass-media coverage, its influence on public awareness of climate-change issues, and implications for Japan’s national campaign to reduce greenhouse gas emissions. Glob. Environ. Change.

[9] Downs A (1972). Up and down with ecology: the ‘issue-attention’ cycle. Public Interest.

[10] Baumgartner, F. & Jones, B. D. *Agendas and instability in American politics*. (University of Chicago, Chicago, 1993).

[11] Gordon JC, Deines T, Havice J (2010). Global warming coverage in the media: trends in a Mexico City newspaper. Sci. Commun..

[12] Takahashi B, Meisner M (2012). Environmental discourses and discourse coalitions in the reconfiguration of Peru's environmental governance. Environ. Commun.: A J. Nat. Cult..

[13] Kingdon JW (2001). A model of agenda-setting, with applications. Law Rev..

[14] Liu X, Lindquist E, Vedlitz V (2011). Explaining media and congressional atttention to global climate change, 1969-2005: an empirical test of agenda-setting theory. Political Res. Q..

[15] Jakob M (2012). Will history repeat itself? Economic convergence and convergence in energy use patterns. Energy Econ..

[16] Gupta, J. *The climate change convention and developing countries: from conflict to consensus*? (Kluwer Academic Publishers, Netherlands, 1997).

[17] UNFCCC. *Nationally Determined Contributions. United Nations Framework Convention on Climate Change*. https://unfccc.int/process-and-meetings/the-paris-agreement/nationally-determined-contributions-ndcs (2019).

[18] Nachmany, M. and Setzer, J. *Global trends in climate change legislation and litigation: 2018 a snapshot. Policy Brief*. (Centre for Climate Change Economics and Policy, Grantham Research Institute on Climate Change and the Environment, U.K., 2018).

[19] Ahchong K, Dodds R (2012). Anthropogenic climate change coverage in two Canadian newspapers, the Toronto Star and the Globe and Mail, from 1988 to 2007. Environ. Sci. Policy.

[20] Gupta J (2010). A history of international climate change policy. WIREs Clim. Change.

[21] Resosudarmo, B. P., Ardiansyah, F. and Napitupulu, L. The Dynamics of Climate Change Governance in Indonesia, in *Climate Governance in the Developing World* (eds Held, D., Roger, Ch. & Nag, E. M.) Ch. 4 (Polity, Cambridge, 2013).

[22] Fletcher, R. Making ´Peace with Nature`: Costa Rica´s Campaign for Climate Neutrality. in *Climate Governance in the Developing World* (eds Held, D., Roger, Ch. & Nag, E. M.) Ch. 8 (Polity, Cambridge, 2013).

[23] Held, D., Roger, Ch. and Nag, E. M. A Green Revolution: China´s Governance of Energy and Climate Change, in *Climate Governance in the Developing World* (eds Held, D., Roger, Ch. and Nag, E. M.) Ch. 2 (Polity, Cambridge, 2013).

[24] Masters, L. Reaching the Crossroads: The Development of Climate Governance in South Africa. in *Climate Governance in the Developing World* (eds Held, D., Roger, Ch. and Nag, E. M.) Ch. 13 (Polity, Cambridge, 2013).

[25] Lee, J. S. Low Carbon Green Growth and Climate Change Governance in South Korea. in *Climate Governance in the Developing World* (eds Held, D., Roger, Ch. & Nag, E. M.) Ch. 5 (Polity, Cambridge, 2013).

[26] Gupta J (2016). Climate change governance: history, future, and triple-loop learning?. WIREs Clim. Change.

[27] Meilstrup, P. The Runaway Summit: The Background Story of the Danish Presidency of COP 15, the UN Climate Change Conference, in *Danish Foreign Policy Yearbook* (eds Hvdit, N. & Mouritzen, H.) 113–135 (Danish Institute for International Studies, 2010).

[28] UNFCCC. *Paris Agreement* (United Nations Framework Convention on Climate Change, New York, 2015).

[29] Raftery AE (2017). Less than 2 °C warming by 2011 unlikely. Nat. Clim. Change.

[30] Nieto J, Carpintero O, Miguel LJ (2018). Less than 2 °C? An economic-environmental evaluation of the Paris Agreement. Ecol. Econ..

[31] Held, D., Roger, C. and Nag, E. M. *Editors´ Introduction: Climate Governance in the Developing World* Ch. 1 (Polity, Cambridge, 2013).

[32] Urrutia, A. and Villanueva, D. *Abolidos, el modelo neoliberal y su política de pillaje, asegura AMLO*. (Periódico La Jornada, Mexico, 2019).

[33] Robles de la Rosa, L. *Mayoría absoluta vuelve al Congreso de la Unión*. (Periódico Excélsior, Mexico, 2018).

[34] Elder Ch, Cobb R (1984). Agenda-building and the politics of aging. Policy Sci. J..

[35] Aguilar Villanueva, L. F. *Problemas Públicos y Agenda de Gobierno* (MA Porrúa, México, 2014).

[36] Riffe, D., Lacy, S. & Fico, F. G. *Analysing Media Messages. Using Quantitative Content Analysis in Research* 2nd edn (Lawrence Erlbaum Associates, Publishers, Mahwah, London, 2005).

[37] NDP. Plan Nacional de Desarrollo 1995-2000, Diario Oficial, 31 de Mayo de 1995 http://www.diputados.gob.mx/LeyesBiblio/compila/pnd/PND_1995-2000_31may95.doc (1995) Accessed 2nd May 2017.

[38] NDP. Plan Nacional de Desarrollo 2001-2006, Diario Oficial, 31 de Mayo de 2001 http://www.diputados.gob.mx/LeyesBiblio/compila/pnd/PND_2001-2006_30may01.doc (2001) Accessed 2nd May 2017.

[39] NDP. Plan Nacional de Desarrollo 2007-2012, Diario Oficial, 31 de Mayo de 2007 http://www.diputados.gob.mx/LeyesBiblio/compila/pnd/PND_2007-2012_31may07.doc (2007) Accessed 2nd May 2017.

[40] NDP. Plan Nacional de Desarrollo 2013-2018, Diario Oficial, 31 de Mayo de 2013 http://www.diputados.gob.mx/LeyesBiblio/compila/pnd/PND_2013-2018_20may13.doc (2013) Accessed 2nd May 2017.

[41] Perezcano-Díaz H (2007). Los tratados internacionales en el orden jurídico Mexicano. Anuario Mexicano de. Derecho Internacional.

[42] Becerra Ramírez, M. et al. Tratados internacionales. Se ubican jerárquicamente por encima de. la leyes y. en. un. segundo plano respecto de. la Constitución Federal (Amparo En Revisión 1475/98). Cuestiones Constitucionales Rev. Mexicana de. Derecho Constitucional **3**, 169–208 (2000).

[43] D. O. F. Ley de Planeación, Nueva Ley Publicada en el Diario Oficial de la Federación, 5 de Enero de 1983, Reforma DOF 16-02-2018, Diario Oficial de la Federación (2018).

[44] Sagasti FR (1988). National development planning in turbulent times: new approaches and criteria for institutional design. World Dev..

[45] Mejia Guinad, L. B. *The Changing Role of the Central Planning Offices in Latin America* Vol 16, 477–491 (Public Organization Review, 2016).

[46] Waterston, A. Development planning: lessons of experience (English) http://documents.worldbank.org/curated/en/299401468767072805/Development-planning-lessons-of-experience (The World Bank, Washington, D.C, 1969).

[47] South. (1986). “Third World Development Plans: Strategies for a decade”. South Mag..

[48] Thorpe A, Reid C, van Anrooy R, Brugere C (2005). When fisheries influence national policy-making: an analysis of the national development strategies of major fish-producing nations in the developing world. Mar. Policy.

[49] Pulver Simone, Sainz-Santamaría Jaime (2017). Characterizing the climate issue context in Mexico: reporting on climate change in Mexican newspapers, 1996–2009. Climate and Development.

[50] UCS. Each´s country share of CO2 Emissions. Union of Concerned Scientists, Available online: https://www.ucsusa.org/global-warming/science-and-impacts/science/each-countrys-share-of-co2.html#.XEtm8M9KjOQ (2018).

[51] Adam, D. México leads the way with carbon reduction pledge. The Guardian, disponible en linea: http://www.theguardian.com/environment/2008/dec/11/poznan-climate-change-mexico-carbon-pledge (2008).

[52] Vance E (2012). México passes climate-change law. Nature.

[53] D. O. F. Decreto por el que se expide la Ley General de Cambio Climático, SEMARNAT, Diario Oficial de la Federación, 6 June 2012, México, D.F. (2012).

[54] Flores VD (1994). El ingreso de México a la OCDE. Rev. de. Comer. Exter..

[55] AP. Mexico formally invited to join OECD as 25th Member. https://apnews.com/5c32c538b21ed50047612bcd16725a5c (Associated Press, 1994).

[56] Geo-México. Why Mexico is in the OECD? Geo-Mexico, The Geography of Mexico. http://geo-mexico.com/?p=8410 (2013).

[57] Selin, H. & VanDeveer, S. D. *Chaning Climates in North American Politics. Institutions Policymaking and Multilevel Governance*. (MIT Press, London, 2009).

[58] Pulver, S. Climate politics in Mexico in a North American Perspective. Presented at Climate Change Politics in North America. (Woodrow Wilson International Center for Scholars, Washington, D.C., 2006).

[59] Pulver, S. “A. Climate Leader? The politics and practice of climate governance in Mexico” in *Climate Governance in the Developing World* (eds D. Held, et al.) 25–46 (MIT Press, Cambridge, 2013).

[60] CICC. Estrategia Nacional de Cambio Climático México. Comisión Intersecretarial de Cambio Climático, Mayo 2007, México, D.F. (2007).

[61] DOF. PROGRAMA Especial de Cambio Climático 2009-2012. SEMARNAT, Diario Oficial de la Federación, 28 de Agosto de 2009, México (2009).

[62] UNFCCC. Report of the conference of the parties on its sixteenth session, held in Cancun from 29 November to 10 December 2010, (United Nations Framework Convention on Climate Change, Bonn, 2011) 15 March 2011.

[63] CONAFOR. Visión de México sobre REDD+: Hacia una estrategia nacional.Comisión Nacional Forestal, Mexico (2010).

[64] DOF. Reglas de Operación del Programa ProArbol 2012. SEMARNAT, Diario Oficial de la Federación, 21 de Diciembre de 2012, México (2011).

[65] Averchenkova, A. & Guzmán-Luna, S. *Mexico´s General Law on Climate Change: Key achievements and challenges ahead*. (Grantham Research Institute on Climate Change and the Environment and the Centre for Climate Change Economics and Policy, London School of Economics and Political Science, London, 2018).

[66] SEMARNAT. Estrategia Nacional de Cambio Climático Visión 10-20-40, Primera Edición, SEMARNAT, México (2013).

[67] CICC. Programa Especial de Cambio Climático (2014-2018). Secretaría de Medio Ambiente y Recursos Naturales. Available online: http://www.semarnat.gob.mx/sites/default/files/documentos/transparencia/programa_especial_de_cambio_climatico_2014-2018.pdf (2014) Accessed 20 March 2019.

[68] DOF. DECRETO por el que se reforman, adicionan y derogan diversas disposiciones de la Ley del Impuesto al Valor Agregado; de la Ley del Impuesto Especial sobre Producción y Servicios; de la Ley Federal de Derechos, se expide la Ley del Impuesto sobre la Renta, y se abrogan la Ley del Impuesto Empresarial a Tasa Única, y la Ley del Impuesto a los Depósitos en Efectivo (Continúa en la Tercera Sección). Diario Oficial de la Federación. 11 de Diciembre de 2013. México, D.F.(2013).

[69] CONAFOR. Estrategia Nacional para REDD+ 2017-2030. ENAREDD+. Comisión Nacional Forestal, Agosto de 2017, México (2017).

[70] SEMARNAT. Programa de pruebas del Sistema de comercio de Emisiones. Secretaria de Medio Ambiente y Recursos Naturales, 27 de Noviembre de 2018. https://www.gob.mx/semarnat/acciones-y-programas/programa-de-prueba-del-sistema-de-comercio-de-emisiones-179414 (2018).

[71] DOF. DECRETO por el que se reforman y adicionan diversas disposiciones a la Ley General de Cambio Climático. SEMARNAT Diario Oficial de la Federación, http://dof.gob.mx/nota_detalle.php?codigo=5531463&fecha=13/07/2018 (2018) Accessed 20 March 2019.

[72] Fenhann, J. CDM Pipeline overview. UNEP-Risoe. http://www.cdmpipeline.org/ (2019).

[73] CICC. México. Sexta Comunicación Nacional y Segundo Informe Bienal de Actualización ante la Convención Marco de las Naciones Unidas sobre el Cambio Climático. Comisión Intersecretarial de Cambio Climático, Noviembre 2018, Mexico. https://unfccc.int/resource/docs/natc/mexnc5s.pdf (2018) Accessed 20 March 2019.

[74] UNFCCC. México y Singapur aceptan la Enmienda de Doha al Protocolo de Kyoto. United Nations Framework Convention on Climate Change. Available on line: https://unfccc.int/es/news/mexico-y-singapur-aceptan-la-enmienda-de-doha-al-protocolo-de-kyoto-1 (2014) Accessed 20th March 2019.

[75] CICC. México. Quinta Comunicación Nacional ante la Convención Marco de las Naciones Unidas sobre el Cambio Climático. Comisión Intersecretarial de Cambio Climático, Noviembre 2012, México. https://unfccc.int/resource/docs/natc/mexnc5s.pdf Accessed 20 March 2019, (2012).

[76] INECC. Evaluación Estratégica del Programa Especial de Cambio Climático 2014-2018. Instituto Nacional de Ecología y Cambio Climático, Secretaría de Medio Ambiente y Recursos Naturales, México. https://www.gob.mx/cms/uploads/attachment/file/261388/Informe__evaluacion_PECC_final_limpio_1_.pdf (2017) Accessed 20 March 2019.

[77] CRE. Aviso para el reporte de Registro Nacional de Emisiones. Factor de emisión por consumo de electricidad, 2014. Comisión Reguladora de Energía, Octubre 2015. http://www.semarnat.gob.mx/sites/default/files/documentos/cicc/aviso_factor_de_emision_electrico.pdf Accessed 20 March, 2019, (2015).

[78] CRE. Factor de emisión del Sector Eléctrico Nacional, 2018. Comisión Reguladora de Energía, Febrero 2019. https://www.gob.mx/cms/uploads/attachment/file/442910/Aviso_Factor_de_Emisiones_2018.pdf (2019).

[79] DOF. Presupuesto de Egresos de la Federación para el Ejercicio Fiscal 2012. Cámara de Diputados H. Congreso de la Unión, Diario Oficial de la Federación, 27 de Diciembre de 2017, (2011).

[80] DOF. Presupuesto de Egresos de la Federación para el Ejercicio Fiscal 2006. Cámara de Diputado H. Congreso de la Unión, Diario Oficial de la Federación, 22 de Diciembre de 2005, (2005).

[81] BANXICO. Cotización de divisas que conforman la canasta del DEG 1/y del DEG respecto al Peso Mexicano 2/ Pesos por Divisa. Sistema de Información Económica. http://www.banxico.org.mx/SieInternet/consultarDirectorioInternetAction.do?accion=consultarCuadroAnalitico&idCuadro=CA91& (2019).

[82] INEGI. Inflación Histórica. Calculadora de Inflación. Instituto Nacional de Estadística y Geografía, Mexico. https://www.inegi.org.mx/sistemas/indiceprecios/CalculadoraInflacion.aspx (2019).

[83] DOF. Presupuesto de Egresos de la Federación para el Ejercicio Fiscal 2018. Cámara de Diputado H. Congreso de la Unión, Diario Oficial de la Federación, 29 de Noviembre de 2017, (2017).

[84] Nakhooda, S. and Norman, M. Climate Finance: is it making a difference? Report. (ODI, London, 2014).

[85] CFU. Climate Funds Update, Overseas Development Institute, Heinrich Boll Stiftung. https://climatefundsupdate.org/ (2019).

[86] Boykoff, M., and Roberts, T. J. Media coverage of climate change: Current trends, strengths, weaknesses. Human Development Reportn2007/2008 Occassional Paper. (UNDP, 2007).

[87] McGregor, J. Three Amigos Summit: What Trudeau, Obama and Pena Nieto agreed on CBC News, 29 June 2016, (2016).

[88] UN. Mexico-President Addresses General, 71st Session, UN Web TV. http://webtv.un.org/watch/mexico-president-addresses-general-debate-71st-session/5133960947001 (2016) Accessed 1 June 2018.

[89] King, E. Patricia Espinosa: Who is the UN’s incoming climate change chief? Climate Home News. https://www.climatechangenews.com/2016/05/05/patricia-espinosa-who-is-the-uns-incoming-climate-change-chief/ (2016) Accessed 30th October 2019.

[90] GOR. I*ntended Nationally Determined Contribution*. (Gobierno de la República, México, 2015).

[91] Chow GC (1960). Tests of equality between sets of coefficients in two linear regressions. Econometrica.

[92] CPC. Debate presidencial 26 de Abril 2006, Comunicación Política y Ciudadania. https://www.youtube.com/watch?v=RTQtE_0kt4I&t=25s. (2015) Accessed 10 November 2018.

[93] CPC. Debate presidencial 6 de Junio 2006, Comunicación Política y Ciudadania. https://www.youtube.com/watch?v=9WOvWxLGVBM&t=78s (2015) Accessed 10 November 2018.

[94] Fernández-Reyes R (2015). Similarities and contrasts between media coverage and Google web search of “climate change” and “global warming” in Spain. Razón y. Palabra.

[95] Stern, N. et al. *Stern Review: The Economics of Climate Change* (HM Treasury, London, 2006).

[96] Vergara-Aceves, J. Acercamiento al nuevo PRI, Análisis Plural, 2º Semestre de 2012. 127–142 (2013).

[97] Boykoff, M., et al. World Newspaper Coverage of Climate Change or Global Warming, 2004-2019. Center for Science and Technology Policy Research, Cooperative Institute for Research in Environmental Sciences, University of Colorado, Media and Climate Change Observatory Data Sets. 10.25810/4c3b-b819 (2019).

[98] Trench, T., Larson, A. M., Libert-Amico A. & Ravikumar, A. *Analyzing multilevel governance in Mexico. Lessons for REDD+ from a astudy of land-use change and benefit sharing in Chiapas and Yucatán, Working Paper 236* (Indonesia Center for International Forestry Research, Bogor, 2018).

[99] CCMSS. *Presupuesto Forestal 2016 ¿Nuevos riesgos para los bosques? Monitoreo de Políticas Públicas* (Nota Informativa 43, Mexico City, 2015).

[100] Sin Embargo. *Elecciones: los temas ausentes* (Sin Embargo, 27 Junio, México, 2012).

[101] Martínez, C. *Destaca propuesta ambiental de AMLO* (Mural, 16 Mayo, 2018).

[102] GRICCE. Climate Change Laws of the World database, Grantham Research Institute on Climate Change and the Environment and Sabin Center for Climate Change Law. http://www.lse.ac.uk/GranthamInstitute/legislation/ (2018).

[103] Townshed T (2013). How national legislation can help to solve climate change. Nat. Clim. Change.

[104] Liu X, Vedlitz A, Alston L (2008). Regional news portrayals of global warming and climate change. Environ. Sci. Policy.

[105] Liu X (2015). Scientists’ views and positions on global warming and climate change: a content analysis of congressional testimonies. Climatic Change.

[106] EZPL. Presidencia de la Republica. Sistema Internet de la Presidencia, Administracion 1994-2000, Enrique Zedillo Ponce de Leon. http://zedillo.presidencia.gob.mx/ (2000).

[107] VFQ. Presidencia de la Republica. Administracion 2000-2006, Vicente Fox Quezada. http://fox.presidencia.gob.mx/ (2006).

[108] FCH. Presidencia de la Republica. Administracion 2000-2012, Felipe Calderon Hinojosa. http://calderon.presidencia.gob.mx/ (2012).

[109] EPN. Presidencia de la Republica. Administracion 2012-2018, Enrique Peña Nieto. https://www.gob.mx/busqueda?utf8=%E2%9C%93&site=presidencia&q= (2017).

[110] AMLO. Administraciones Anteriores. Presidencia de la República. http://www.gob.mx/presidencia/acciones-y-programas/administraciones-anteriores (2019).

[111] Kirp DL (1982). Professionalization as a policy choice. World Politics.

[112] Howell, D. C. *Statistical Methods for Psychology*. (Wadsworth CENGE Learning, USA, 2010).

