# Supplementary material for: The systemic and governmental agendas in presidential attention to climate change in Mexico 1994–2018
Source: Nat Commun. 2020 Jan 23;11:455. doi: 10.1038/s41467-019-14048-7 (PMC6978510; doi:10.1038/s41467-019-14048-7)
Supplement: Supplementary file 1 — Description of Additional Supplementary Files [file 41467_2019_14048_MOESM1_ESM.pdf]

Description of Additional Supplementary Files

File Name: Supplementary Data 1

Description: Database of presidential communications referring to climate change in Mexico 1994-2018.
