# Supplementary material for: The systemic and governmental agendas in presidential attention to climate change in Mexico 1994–2018
Source: Nat Commun. 2020 Jan 23;11:455. doi: 10.1038/s41467-019-14048-7 (PMC6978510; doi:10.1038/s41467-019-14048-7)
Supplement: Supplementary file 2 — Supplementary Data 1 [file 41467_2019_14048_MOESM2_ESM.pdf]

Date	Title
01/09/95	Primer Informe: Medio Ambiente, Recursos Naturales y Pesca
01/09/95	Primer Informe: Política Exterior
01/11/97	Declaración de líderes económicos de APEC:Conectando la comunidad de APEC
14/11/97	Declaración de los presidentes Ernesto Zedillo y William Clinton
14/11/97	Palabras del presidente Ernesto Zedillo, durante el almuerzo que le ofreció el Excmo. Albert Gore, vicepresidente de los Estados Unidos de América, hoy en la tarde en el salón "Benjamin Franklin" del edificio del Departamento de Estado, en esta ciudad.
14/11/97	Versión estenográfica de las palabras del Excmo. señor William Clinton, presidente de los Estados Unidos de América, durante la ceremonia a la que asistió el presidente Ernesto Zedillo, con motivo de la firma de la *Convención Interamericana Contra la Fabricación y el Tráfico Ilícito de Armas de Fuego, Municiones, Explosivos y Otros*, hoy al mediodía, en el Salón de Las Américas, de la Organización de Estados Americanos, en su sede (17 Street y Constitution Avenue), en esta ciudad.
24/11/97	Versión estenográfica de la conferencia de prensa que ofreció el licenciado José Angel Gurría Treviño, secretario de Relaciones Exteriores, hoy en la noche, en la Sala de Prensa del hotel *Landmark*, en esta ciudad.
23/03/98	Versión estenográfica de las palabras del presidente Ernesto Zedillo, durante la comida que encabezó con integrantes de la Concamín, en la que declaró clausurada la LXXX Asamblea General Ordinaria de dicho organismo, en esta ciudad.
01/04/98	Plan de Acción II Cumbre de las Américas
01/04/98	Versión estenográfica de las palabras del presidente Ernesto Zedillo, durante la ceremonia en la que la M. en C. Julia Carabias Lillo, presentó el Programa de Trabajo de la Secretaría de Medio Ambiente, Recursos Naturales y Pesca 1998, en el Parque Ecológico "Diego Muñoz Camargo", perteneciente a este municipio
19/04/98	Declaración de Santiago
19/04/98	VII Reunión Cumbre del Grupo de Río
09/06/98	Cumbre Mundial Contra las Drogas
09/06/98	México suscribió el Protocolo de Kioto sobre Cambio Climático, en la sede de la ONU
17/07/98	DECLARACION CONJUNTA DE LA III REUNION DE JEFES DE ESTADO Y DE GOBIERNO DE LOS PAISES INTEGRANTES DEL MECANISMO DE DIALOGO Y CONCERTACION DE TUXTLA
15/01/99	Visita de Estado a la República de Costa Rica del doctor Ernesto Zedillo Ponce de León, presidente de los Estados Unidos Mexicanos
12/02/99	Versión estenográfica del briefing que ofreció el licenciado Fernando Lerdo de Tejada, director general de Comunicación Social y vocero de la Presidencia de la República, en la sala de prensa de la residencia oficial de Los Pinos.
12/02/99	Cobertura Especial de la Visita de Trabajo de William J. Clinton, Presidente de los E.U.A., a la ciudad de Mérida, Yucatán.
15/02/99	Versión estenográfica del briefing que ofreció el licenciado Fernando Lerdo de Tejada, director general de Comunicación Social y vocero de la Presidencia de la República, en la sala de prensa de la residencia oficial de Los Pinos.
15/02/99	Versión estenográfica de las palabras del presidente Ernesto Zedillo, durante la ceremonia de Firma de Acuerdos y Declaración Conjunta entre México y Estados Unidos de América, que encabezó con el presidente William J. Clinton, en el teatro "José Peón Contreras", en esta ciudad.
15/02/99	Versión estenográfica de las palabras del presidente William J. Clinton, durante la ceremonia de la Firma de Acuerdos y Declaración Conjunta que encabezó con el presidente Ernesto Zedillo, en el teatro "José Peón Contreras", en esta ciudad.
29/03/99	Protección integral de los bosques y el medio ambiente
29/03/99	Versión estenográfica de las palabras del presidente Ernesto Zedillo, durante el Foro Internacional sobre el uso del Fuego en las Actividades Agropecuarias y Forestales, en la Hondonada, de la residencia oficial de Los Pinos.
29/06/99	DECLARACIÓN DE RÍO DE JANEIRO
11/10/99	Versión estenográfica de la entrevista que concedió el presidente Ernesto Zedillo a los medios de comunicación, al término de su recorrido que hizo por zonas que afectaron las lluvias en los estados de Puebla y Veracruz.
28/01/00	Versión estenográfica de las palabras del presidente Ernesto Zedillo, durante la Sesión Plenaria de la Reunión Anual, 2000, del Foro Económico Mundial, en el auditorio del Centro de Congresos, de esta ciudad.
03/05/00	Versión estenográfica de las palabras del presidente Ernesto Zedillo, durante la presentación del Programa de Rehabilitación de la Laguna de Cuyutlán, en la que hizo entrega de las obras de dragado y escolleras, en Boca de Tepalcates, de este puerto.
02/10/00	Cooperación entre naciones, única vía de solución al riesgo ambiental
02/10/00	Cooperación entre naciones, única vía de solución al riesgo ambiental

Date	Title
02/10/00	Versión estenográfica de las palabras del presidente Ernesto Zedillo, durante la Reunión de Ministros de Medio Ambiente de Latinoamérica y el Caribe, que encabezó en el salón Manuel Avila Camacho, de la residencia oficial de Los Pinos.
16/08/01	Vicente Fox durante la Cena de Estado que le ofreció el Presidente Ricardo Lagos Escobar
13/11/01	Declaración Conjunta suscrito por el Presidente Fox y la Primera Ministra de Nueva Zelandia
15/11/01	Diversas Intervenciones durante la inauguración del Foro Internacional de Federalismo. Parte II
14/05/02	Mensaje del Presidente Fox ante el Pleno del Senado de Bélgica
17/05/02	Cena de Gala ofrecida por Sus Majestades el Rey Juan Carlos I y su esposa, la Reina Sofía
16/08/02	Clausura de los Trabajos del Comité Nacional Preparatorio de la Cumbre Mundial sobre el Desarrollo Sostenible
20/08/02	Defenderá México patrimonio ambiental
26/08/02	Inicia Cumbre de Johannesburgo
28/08/02	Conferencia de prensa sobre la posición de México en la Cumbre de Johannesburgo y resumen de los objetivos de la gira a Nigeria
01/09/02	México logra el inicio de negociaciones para determinar reglas internacionales que regulen el acceso a recursos genéticos
03/09/02	Foro Financiamiento e Inversión para el Desarrollo Sostenible en América Latina y el Caribe
03/09/02	Palabras del Presidente Fox en el Pleno de la Cumbre Mundial sobre Desarrollo Sostenible
04/09/02	Promueve Vicente Fox desarrollo sostenible
07/09/02	Versión estenográfica del Programa Radiofónico Fox Contigo, un programa de la Coordinación de Imagen de la Presidencia de la República, que condujo este mediodía el Presidente Vicente Fox Quesada.
14/11/02	Palabras del Presidente Fox durante la Sesión Solemne de la Asamblea Nacional
27/01/03	Palabras del Presidente Vicente Fox durante la Sesión Especial de Senadores y Representantes del Parlamento Neerlandés
17/02/03	El Presidente Fox durante la inauguración del Seminario Medio Ambiente y Desarrollo: Enfoque Integral del Manejo Sustentable de Recursos Naturales
17/02/03	Por una política de protección ambiental
22/05/03	Declaración Conjunta de interés bilateral para México y el Perú
11/05/04	El Gobierno Federal, preparado para la temporada de lluvias y ciclones tropicales 2004: Presidente Vicente Fox
11/05/04	Palabras del Presidente Vicente Fox durante la 1a Sesión Ordinaria de 2004 del Consejo Nacional de Protección Civil
29/05/04	Declaración de Guadalajara
03/06/04	Palabras del Presidente Vicente Fox durante la ceremonia de inauguración del Coloquio Globalización y Justicia Internacional
07/09/04	Recibe el Presidente Vicente Fox al Secretario General de la ONU, Kofi Annan en Los Pinos
25/10/04	Firma de Acuerdos entre el Gobierno de México y Canadá
16/02/05	Encabeza Presidente Vicente Fox la puesta en marcha en México del Protocolo de Kyoto
16/02/05	México es de las primeras naciones del Continente en ratificar el Protocolo de Kyoto: Vicente Fox Quesada
17/02/05	México, de los primeros en ratificar Protocolo de Kyoto
11/03/05	Cumple México anticipadamente compromisos ambientales: Presidente Vicente Fox
11/03/05	Diversas intervenciones durante la Firma del Compromiso Nacional por la Década de la Educación para el Desarrollo Sustentable
11/03/05	Firma del Compromiso Nacional por la Década de la Educación para el Desarrollo Sustentable
23/03/05	Conferencia de prensa conjunta que ofrecieron los Presidentes de México Vicente Fox, Estados Unidos George W. Bush y el Primer Ministro de Canadá Paul Martin
09/05/05	Diversas intervenciones durante la ceremonia de entrega del Premio México de Ciencia y Tecnologìa 2004
05/06/05	Diversas intervenciones durante la Ceremonia Conmemorativa del Día Mundial del Medio Ambiente
09/06/05	Diversas intervenciones durante la ceremonia de Lanzamiento del Pacto Mundial en México
05/07/05	Participará el Presidente Vicente Fox Quesada en la Reunión del G-8
06/07/05	La participacion de México en la Reunión del G-8, un reconocimiento a su capacidad de interlocución
07/07/05	Concluye Presidente Vicente Fox Quesada su participación en las Reuniones del G-8 y G-5
07/07/05	Conferencia de prensa que concedió el Presidente Vicente Fox Quesada en el marco de la Reunión de los Líderes del G-8
09/07/05	Programa radiofónico Fox Contigo del 9 de julio de 2005
22/08/05	Diversas intervenciones durante el evento Objetivos del Desarrollo del Milenio: Una Mirada Desde América Latina y el Caribe
19/09/05	Diversas intervenciones durante la Ceremonia Conmemorativa del Día Nacional de Protección Civil

Date	Title
19/09/05	Los sismos del 85, gestaron una nueva sociedad que cambió el rumbo de México: Presidente Vicente Fox
30/09/05	Declaración Conjunta México-Canadá
30/09/05	Conferencia de Prensa Conjunta ofrecida por el Presidente Fox y el Primer Ministro de Canadá, Paul Martin
15/10/05	Programa Radiofónico Fox Contigo del 15 de octubre de 2005
17/10/05	El Presidente Fox dedicará esta semana a las comunidades indígenas, en el marco de los 50 municipios más pobres del país: Rubén Aguilar, Vocero de Presidencia
13/12/05	Diálogo que sostuvo el Presidente Vicente Fox durante la inauguración de la Cumbre sobre la Iniciativa Energética Mesoamericana. Segunda parte
10/03/06	El fondo de las PyMES ha impulsado a más de 20 mil empresas, y los beneficiarios han sido 50 mil emprendedores: Rubén Aguilar, Vocero de Presidencia
11/03/06	Programa radiofónico Fox Contigo del 11 de marzo de 2006
14/03/06	"Nunca en la historia de este país en un sexenio se había construido una cantidad tan importante de generación eléctrica": Alfredo Elías, director general de la CFE
15/03/06	México, sede del IV Foro Mundial del Agua
22/05/06	Tenemos una cobertura de 96.5 por ciento de la población mexicana con energía eléctrica, uno de los índices más altos a nivel mundial: Fernando Canales, Secretario de Energía
05/06/06	Celebracion del Día Mundial del Medio Ambiente
24/07/06	Diversas intervenciones durante el Inicio del llenado del Embalse de la Presa El Cajón
26/07/06	Ceremonia de inauguración del Campo Geotérmico Los Azufres II
26/07/06	Diversas intervenciones durante la Ceremonia de inauguración del Campo Geotérmico Los Azufres II
08/08/06	Diversas intervenciones durante la Toma de Protesta al Consejo Nacional Directivo de la Cámara Nacional del Autotransporte de Pasaje y Turismo, CANAPAT, 2006-2007
16/08/06	Diversas intervenciones durante la Ceremonia del 69 Aniversario de la creación de la Comisión Federal de Electricidad
17/08/06	La educación ambiental es un tema en el que hemos participado muy activamente: José Luis Luege Tamargo, Secretario de la SEMARNAT. Primera Parte
13/09/06	Diversas intervenciones durante la Visita a la Planta de Camiones y Motores International de México, S.A. de C.V.
18/09/06	La Gira a Nueva York del Presidente Fox, tiene seis objetivos: Rubén Aguilar, Vocero de Presidencia
04/10/06	Recibe en Los Pinos el Presidente Vicente Fox Quesada a Ministra de Asuntos Exteriores del Reino Unido
20/10/06	Diversas intervenciones durante la ceremonia del Lanzamiento de la Gasolina PEMEX Premium Ultra Bajo Azufre
20/10/06	El Presidente Vicente Fox hará el lanzamiento oficial de la Gasolina Premium Ultra Bajo Azufre: José Luis Luege, Secretario de SEMARNAT
29/01/07	Declaración conjunta del Presidente de México y del Primer Ministro del Reino Unido
29/01/07	El Presidente Felipe Calderón en la Reunión sobre el Desarrollo Sustentable y Cambio Climático
20/02/07	Diversas intervenciones durante la Presentación Nacional del Programa PROÁRBOL
20/02/07	El Presidente Calderón en la Presentación Nacional del Programa PROÁRBOL
21/02/07	Diversas intervenciones durante el evento Estrategia Nacional para el Ordenamiento Ecológico del Territorio en Mares y Costas
21/02/07	El Presidente Calderón durante la presentación de la Estrategia Nacional para el Ordenamiento Ecológico del Territorio en Mares y Costas
24/02/07	El Presidente Calderón en el evento Compromisos por la Conservación
28/02/07	El Presidente Calderón en la Inauguración del Fraccionamiento "Bosques de Ciudad Tres Marías"
09/03/07	El Presidente Calderón en el evento Fortalecimiento del Programa Joven Emprendedor Rural-Fondo de Tierras y Programa de Modernización y Actualización del Catastro Rural
14/03/07	Diversas intervenciones durante la Campaña Nacional de Prevención y Combate de Incendios Forestales
14/03/07	El Presidente Calderón en la Campaña Nacional de Prevención y Combate de Incendios Forestales
15/03/07	El Presidente Calderón en la Presentación del Balance de Inicio de Gobierno
16/03/07	El Presidente Calderón en la Inauguración de la Planta de Aguas Residuales "Las Arenitas"
16/03/07	El Presidente Calderón en la Presentación del Proyecto Valle Las Palmas, Modelo de Desarrollo de una Ciudad Sustentable
18/03/07	El Presidente Calderón en la Ceremonia Conmemorativa del LXIX Aniversario de la Expropiación de la Industria Petrolera
22/03/07	Diversas intervenciones en la Inauguración de la Presa de Almacenamiento "Barreto"
22/03/07	El Presidente Calderón en la ceremonia de Inauguración de la Presa de Almacenamiento "Barreto"
27/03/07	El Presidente Calderón en la Inauguración del V Foro México Siglo XXI "Jóvenes Construyendo"-Fundación TELMEX
29/03/07	Diversas intervenciones en la Inauguración de la Central Eólica "La Venta II"

Date	Title
29/03/07	El Presidente Calderón en la Inauguración de la Central Eólica “La Venta II”
19/04/07	El Presidente Calderón en la Inauguración de la Planta Desaladora de Los Cabos
22/04/07	Diversas intervenciones en la conmemoración del Día de la Tierra
22/04/07	El Presidente Calderón en la Conmemoración del Día de la Tierra
03/05/07	El Presidente Calderón en la Celebración del Día de la Santa Cruz con Trabajadores de la Industria de la Construcción
17/05/07	El Presidente Calderón en la Entrega de Áreas Naturales Protegidas
21/05/07	El Presidente Calderón en la Presentación del Proyecto Visión 2030, “El México que Queremos”
22/05/07	Diversas intervenciones durante el Día Internacional de la Biodiversidad
22/05/07	El Presidente Calderón en el Día Internacional de la Biodiversidad
23/05/07	Diversas intervenciones en la Presentación del Proyecto Parque Ecológico del Bicentenario
25/05/07	Diversas intervenciones en el evento Estrategia Nacional de Cambio Climático
25/05/07	El Presidente Calderón en la Estrategia Nacional de Cambio Climático
30/05/07	El Presidente Calderón en la 16ª Mesa Redonda con el Gobierno de México: Consenso, Estabilidad y Crecimiento
30/05/07	La Subsecretaria de Relaciones Exteriores, María de Lourdes Aranda Bezaury, con motivo de la Gira de Trabajo por Europa que realizará el Presidente Calderón
31/05/07	Diversas intervenciones en la Presentación del Plan Nacional de Desarrollo
31/05/07	El Presidente Calderón en la Presentación del Plan Nacional de Desarrollo
05/06/07	Conferencia de prensa que ofreció el Presidente Calderón al término de la Cena de Trabajo con Nicolas Sarkozy, Presidente de la República Francesa
05/06/07	El Presidente Calderón inicia Visita de Trabajo al Reino de Bélgica
06/06/07	El Presidente Calderón en la reunión que sostuvo con los Miembros de la Comisión Europea
07/06/07	Conferencia de prensa que ofreció el Presidente Calderón en Berlín, Alemania
08/06/07	Declaración Conjunta de la Presidencia Alemana del G8 y los Jefes de Estado y de Gobierno del G-5 en ocasión de la Cumbre del G8
08/06/07	Declaración de la posición conjunta del G-5 y Participación en la Cumbre del G8
08/06/07	El Presidente Calderón inicia actividades en Dinamarca
08/06/07	Intervención del Presidente Calderón en la Cumbre del Grupo de los 8
08/06/07	Mensaje ofrecido por el Presidente Calderón Hinojosa en Copenhaguen, Dinamarca
22/06/07	El Presidente Calderón en la Entrega de la Presea Lázaro Cárdenas
26/06/07	El Presidente Calderón en la Entrega de la Obra del Entubamiento del Gran Canal del Desagüe al Gobierno del Distrito Federal
27/06/07	El Presidente de Nicaragua, Daniel Ortega Saavedra en la Ceremonia Oficial de Bienvenida que ofreció en su honor el Presidente Calderón
12/07/07	Diversas intervenciones en el Centenario del Vivero Coyoacán 1907-2007
18/07/07	Diversas intervenciones en la Presentación del Programa Nacional de Infraestructura
31/07/07	Mensaje del Presidente Calderón al término de la reunión privada que sostuvo con el señor Albert Gore, Ex vicepresidente de los Estados Unidos de América
06/08/07	Conferencia de Prensa Conjunta que ofrecieron el Presidente Calderón y el Presidente de la República Federativa de Brasil, Luiz Inácio Lula da Silva
09/08/07	Diversas intervenciones en el evento Reforestación Social, Entrega de Recursos PROÁRBOL y Presentación del Programa Biodiversidad
09/08/07	El Presidente Calderón en la Entrega de Recursos PROÁRBOL y Presentación del Programa Biodiversidad
10/08/07	El Presidente Calderón en la Reunión de Trabajo con el Grupo Parlamentario del Partido Acción Nacional del Senado de la República
14/08/07	Diversas intervenciones en la Conmemoración del 70 Aniversario de la Comisión Federal de Electricidad
14/08/07	El Presidente Calderón en la Conmemoración del 70 Aniversario de la Comisión Federal de Electricidad
23/08/07	Mensaje del Presidente Calderón en su recorrido por las zonas afectadas de Tulancingo
02/09/07	Mensaje del Presidente Calderón con motivo del Primer Informe de Gobierno
06/09/07	Conferencia de Prensa Conjunta que ofrecieron el Presidente Calderón y la Primer Ministro de Nueva Zelandia, Helen Clark
19/09/07	Diversas intervenciones durante el evento Día Nacional de Protección Civil
20/09/07	Palabras del Presidente Calderón en la Ceremonia de Inauguración del Fórum Universal de las Culturas Monterrey 2007
21/09/07	Diversas intervenciones durante la Inauguración de la Exposición América Migración
27/09/07	Diversas intervenciones durante la XXV Conferencia de Gobernadores Fronterizos
27/09/07	Diversas intervenciones en la Inauguración de la V Expo Forestal
01/10/07	Diversas intervenciones en la Ceremonia Conmemorativa del XX Aniversario del Protocolo de Montreal
01/10/07	El Presidente Calderón en la Ceremonia Conmemorativa del XX Aniversario del Protocolo de Montreal

Date	Title
02/10/07	El Presidente Calderón en la Inauguración de la Antena de Recepción de Información Satelital
05/10/07	El Presidente Calderón en la comida con motivo de la Clausura del VII Congreso Internacional de Turismo
12/10/07	El Presidente Calderón felicita a Al Gore y al IPCC de la ONU por el Premio Nóbel de la Paz
22/10/07	Diversas intervenciones en la Entrega de Premios de Investigación de la Academia Mexicana de Ciencias
08/11/07	El Presidente Calderón en la Presentación del Programa de Sustentabilidad Hídrica de la Cuenca del Valle de México
27/11/07	Diversas intervenciones en el Mensaje y Comida durante el Primer Encuentro Nacional de Competitividad
28/11/07	Diversas intervenciones durante la Presentación del Programa Sectorial Económico
29/11/07	Diversas intervenciones en la Presentación del Programa Sectorial Social
29/11/07	El Presidente Calderón en la Presentación del Programa Sectorial Social
30/11/07	Diversas intervenciones durante el Anuncio del Inicio de Producción del Primer Vehículo Híbrido producido en México por el Complejo Automotriz General Motors
03/12/07	El Presidente Calderón durante la Conferencia de Prensa que ofreció en la Residencia Oficial.
07/01/08	El Presidente Calderón en el marco de la Reunión de Embajadores y Cónsules de México en el Extranjero
17/01/08	El Presidente Calderón en el evento PROÁRBOL 2008
22/01/08	El Presidente Calderón en el Inicio de Obras de la Presa La Yesca
24/01/08	Diversas intervenciones en la Inauguración de la Pista de Pruebas en Clima Caliente de General Motors México
13/02/08	El Presidente Calderón en la sesión conjunta de la Asamblea de California
18/02/08	Diversas intervenciones en la Ceremonia Magna Inaugural del 50 Aniversario del Instituto Mexicano de Ingenieros Químicos, A.C.
11/03/08	El Presidente Calderón en la Ceremonia Oficial de Bienvenida al Presidente de la República de Islandia, Ólafur Ragnar Grímsson
24/03/08	Diversas intervenciones en la ceremonia de Presentación del Programa Nacional Hídrico 2007-2012
16/04/08	Diversas intervenciones en la Clausura de la 33 Edición del Tianguis Turístico
22/04/08	Declaración Conjunta del Presidente Bush, Presidente Calderón, Primer Ministro Harper en la Cumbre de Líderes de América del Norte
22/04/08	Intervenciones de George W. Bush, Presidente de los Estados Unidos de América, y del Stephen Harper, Primer Ministro de Canadá, en la Conferencia de Prensa Conjunta de los Líderes de América del Norte
29/04/08	Diversas intervenciones en la presentación de la Estrategia de Conservación para el Desarrollo
12/05/08	Recibe el Presidente Calderón a Directivos de Greenpeace
17/05/08	El Presidente se reunió con Jan Peter Balkenende, Primer Ministro de Países Bajos
23/05/08	El Presidente Calderón en el Programa para el Saneamiento Integral de la Bahía de Acapulco
28/05/08	El Presidente Calderón en la Cumbre Cambio Climático y Medio Ambiente, Centroamérica y el Caribe
28/05/08	Intervención del Presidente de El Salvador en la Cumbre Cambio Climático y Medio Ambiente, Centroamérica y el Caribe
28/05/08	Intervención del Presidente de Honduras en la Cumbre Cambio Climático y Medio Ambiente, Centroamérica y el Caribe
28/05/08	La Reforma Energética es constitucional: Felipe Calderón
28/05/08	Llama Felipe Calderón a combatir el cambio climático y la pobreza y desigualdad mundial
28/05/08	Mensaje del Presidente Calderón al término de la reunión con su Homólogo de Guatemala
05/06/08	Diversas intervenciones en la Celebración del Día Mundial del Medio Ambiente
05/06/08	El Presidente Calderón en la Celebración del Día Mundial del Medio Ambiente
05/06/08	Reconoce el Fondo Mundial para la Naturaleza papel estratégico de México contra Cambio Climático
14/06/08	El Presidente Calderón en la Ceremonia Conmemorativa del Día de México en la Expo-Zaragoza 2008
24/06/08	Diversas intervenciones en el Lanzamiento del Programa Piloto de Sustitución de Equipos Electrodomésticos para el Ahorro de Energía
05/07/08	Diversas intervenciones en el evento Planta un árbol y Sé Parte de la historia en el estado de Michoacán
05/07/08	El Presidente Calderón en el evento Planta un Árbol y Sé Parte de la Historia en Michoacán
10/07/08	El Presidente Calderón en el Seminario de Oportunidades de Inversión y Negocios
15/07/08	Diversas intervenciones en el Inicio de Obras de la Terminal de Gas Natural de Manzanillo
15/07/08	El Presidente Felipe Calderón en el Inicio de Obras de la Terminal de Gas Natural de Manzanillo
17/07/08	El Presidente Calderón en la Presentación del Programa de Calidad del Aire para Vivir Mejor
04/08/08	El Presidente Calderón en la Reunión de Trabajo sobre los Objetivos de Desarrollo del Milenio y del Cambio Climático

Date	Title
04/08/08	Mensaje a los medios del Presidente Calderón que tuvo lugar en la Escalinata de la Casa Miguel Alemán
04/09/08	El verdadero desarrollo humano sustentable sólo puede entenderse respetando el medio ambiente: Presidente Calderón
17/09/08	Diversas intervenciones en el Arranque de la ampliación del proyecto Monterrey II de cogeneración de energía eléctrica
17/09/08	El Presidente Calderón en el Arranque de la Ampliación del Proyecto Monterrey II de Cogeneración de Energía Eléctrica
22/09/08	Diversas intervenciones en el evento de Educación Ambiental para Vivir Mejor
22/09/08	El Presidente Calderón en el evento de Educación Ambiental para Vivir Mejor
22/09/08	Elogio de Achim Steiner, Director Ejecutivo del PNUMA, por el compromiso de México con el medio ambiente
23/09/08	El Presidente Calderón en la Cena con los Integrantes de la Foreign Policy Association
24/09/08	El Presidente Calderón en la 63 Asamblea General de la Organización de las Naciones Unidas
24/09/08	EL PRESIDENTE CALDERÓN Y EL PRIMER MINISTRO RASMUSSEN COINCIDEN EN IMPULSAR LOS ESFUERZOS PARA COMBATIR EL CAMBIO CLIMÁTICO
25/09/08	Refrenda el Presidente Calderón la propuesta de constituir el Fondo Verde contra el cambio climático
14/10/08	El Presidente Calderón en el Recorrido por las Áreas Afectadas por el Huracán Norbert en Baja California Sur
29/10/08	Diversas intervenciones durante la XIX Asamblea General Ordinaria y Toma de Protesta de la Cámara Nacional del Autotransporte de Carga
22/11/08	El Presidente Calderón fortalece vínculos de México con Australia, uno de nuestros principales socios en Asia-Pacífico
25/11/08	El Presidente Calderón en su participación en la Sesión Solemne en el Congreso de la Nación
03/12/08	Diversas intervenciones en la Entrega Simbólica del Lote de Autotanques de la Flota de Reparto de Pemex-Refinación
17/12/08	Declaración de la Cumbre de América Latina y el Caribe
05/01/09	El Presidente Calderón en la Inauguración del tramo Lechería-Cuautitlán del Tren Suburbano
05/01/09	Inaugura el Presidente Calderón tramo Lechería-Cuautitlán del Tren Suburbano
09/01/09	El Presidente Calderón en la Comida de Trabajo con motivo de la XX Reunión de Embajadores y Cónsules de México
22/01/09	Diversas intervenciones durante la Inauguración de la Central de Energía Eólica, Parques Ecológicos de México
22/01/09	El Presidente Felipe Calderón en la Inauguración de la Central de Energía Eólica, Parques Ecológicos de México
28/01/09	Conferencia de Prensa que ofreció el Presidente Calderón
29/01/09	El Secretario General de Naciones Unidas reconoce al Presidente Calderón la labor proactiva de México en materia de cambio climático
29/01/09	Refrenda el Presidente Calderón compromiso de México con el impulso a un desarrollo global sustentable
30/01/09	Actividades de Margarita Zavala en el Marco del Foro Económico de Davos, Suiza
05/03/09	Diversas intervenciones en la Inauguración de la Expansión de la Empresa Kyocera Mexicana S.A de C.V
09/03/09	El Presidente Calderón en la Firma de documentos y mensaje conjunto a medios de comunicación
10/03/09	Diversas intervenciones en el evento de Encuentro logros y avances de la Fundación Alfredo Harp Helú, A.C.
11/03/09	El Presidente Calderón en la Yesca: Desvío del Río Santiago e Inicio de Construcción de la Cortina
19/03/09	El Presidente Calderón en el Almuerzo en Honor de Sus Altezas Reales Haakon y Mette-Marit, Príncipes Herederos de Noruega
19/03/09	Intervención de Su Alteza Real, Príncipe Haakon Magnus, Príncipe Heredero de Noruega, en el Almuerzo que ofreció en su honor el Presidente Calderón
19/03/09	México y Noruega comparten retos y oportunidades: Presidente Calderón
26/03/09	Diversas intervenciones durante el Lanzamiento del Programa Nacional de Sustitución de Electrodomésticos
26/03/09	Palabras del Presidente Felipe Calderón en el Lanzamiento del Programa Nacional de Sustitución de Electrodomésticos
31/03/09	El Presidente Calderón en la Cena en su honor que ofrece el Lord Mayor de la City, acompañado por miembros de la Familia Real
01/04/09	El cambio climático, uno de los retos más grandes que enfrenta la humanidad
01/04/09	El Presidente Calderón en la Participación en el 75 Aniversario del Consejo Británico
01/04/09	El Presidente Calderón en su Intervención ante integrantes de ambas Cámaras del Parlamento del Reino Unido
02/04/09	El Presidente Calderón en el mensaje a medios de comunicación mexicanos

Date	Title
14/04/09	Diversas intervenciones en la Presentación del Programa Nacional para la Prevención y Gestión Integral de Residuos
14/04/09	El Presidente Calderón en la Presentación del Programa Nacional para la Prevención y Gestión Integral de Residuos
17/04/09	Se reúne el Presidente Calderón con el Primer Ministro de Canadá, Stephen Harper, en la V Cumbre de las Américas
22/04/09	Diversas intervenciones en la Conmemoración del Día Internacional de la Tierra
22/04/09	El Gobierno Federal no cederá ante depredadores de la tierra
22/04/09	El Presidente Calderón en la Conmemoración del Día Internacional de la Tierra
23/04/09	Diversas intervenciones en el Lanzamiento del Programa Nacional de Sustitución de Electrodomésticos
23/04/09	El Presidente Calderón en el Lanzamiento del Programa Nacional de Sustitución de Electrodomésticos
05/06/09	Anuncia el Presidente Calderón Programa Especial contra el Cambio Climático
05/06/09	Diversas intervenciones en el Día Mundial del Medio Ambiente 2009
05/06/09	El Presidente Calderón en el Día Mundial del Medio Ambiente 2009
17/06/09	El Presidente Calderón en la Instalación del Consejo Estatal de Protección Civil, Tapachula Chiapas
17/06/09	Prioritario proteger la vida, integridad y patrimonio de los mexicanos ante desastres naturales
22/06/09	Diversas intervenciones en la Tercera Reunión Preparatoria del Foro las Grandes Economías Sobre Energía y Clima
22/06/09	El Presidente Calderón en la Tercera Reunión Preparatoria del Foro las Grandes Economías Sobre Energía y Clima
22/06/09	Presidente Calderón, socio vital en la lucha contra el cambio climático
08/07/09	Declaración Política del G-5
08/07/09	El Presidente Calderón en el Mensaje Conjunto de los Jefes de Estado y/o de Gobierno del G5 a los Medios de Comunicación
09/07/09	Informe Final sobre el Proceso de Heiligendamm
09/07/09	Se reúne el Presidente Felipe Calderón con el Primer Ministro de Suecia, Fredrik Reinfeldt
10/07/09	El cambio climático es uno de los mayores desafíos de nuestro tiempo tenemos la intención de responder enérgicamente a este reto: Líderes Foro de las Principales Economías sobre Energía y Clima
06/08/09	Diversas intervenciones en la Inauguración de las Plantas Criogénicas Modulares 5 y 6 en el Complejo Procesador de Gas Burgos
09/08/09	Dialogan el Presidente Calderón y su homólogo estadounidense sobre temas económicos, migratorios y de seguridad bilateral
10/08/09	Declaración de los Líderes de América del Norte sobre Cambio Climático y Energía Limpia
14/08/09	Mensaje de Presidente Felipe Calderón en la Sesión Solemne de la Asamblea General
02/09/09	Mensaje del Presidente Calderón con motivo del Tercer Informe de Gobierno
04/09/09	Mensaje a la Nación del Presidente Calderón en Materia de Medio Ambiente en el marco de su Tercer Informe de Gobierno
07/09/09	Diversas intervenciones en el Mensaje a Medios del Presidente Calderón
10/09/09	El Presidente Calderón en la Comida dentro del Marco del Primer Foro Internacional de Vivienda Sustentable
17/09/09	Diversas intervenciones en la Fundación del Desarrollo Habitacional Nuevo Juan de Grijalva
24/09/09	El Presidente Felipe Calderón en la Sesión del Debate sobre el Desarme y No Proliferación, de los Jefes de Estado y de Gobierno del Consejo de Seguridad de Naciones Unidas
25/09/09	Urgió el Presidente Calderón a la acción comprometida y compartida por todas las naciones en la lucha contra los efectos del cambio climático
26/09/09	El Presidente Calderón en la Clausura de la VII Expo Forestal México Siglo XXI
29/09/09	Reconoció Al Gore liderazgo del Presidente Calderón en las negociaciones de cambio climático
05/10/09	Diversas intervenciones en la Presentación del Informe de Desarrollo Humano 2009
05/10/09	El Presidente Calderón en la Presentación del Informe de Desarrollo Humano 2009
07/10/09	Diversas intervenciones durante la Inauguración del Foro Global de Energías Renovables
07/10/09	El Presidente Calderón en la Inauguración del Foro Global de Energía Renovable
07/10/09	Refrenda Presidente Calderón compromiso de impulsar energías renovables a escala mundial
14/10/09	Diversas intervenciones en la Inauguración de la Exposición Temporal Cambio Climático
14/10/09	El Presidente Calderón en la Inauguración de la Exposición Temporal Cambio Climático
14/10/09	Importante formar a nuevas generaciones en una conciencia ambiental: Presidente Calderón
19/10/09	Diversas intervenciones en el Lanzamiento de la Campaña: El agua es como de tu familia, protégela
19/10/09	El Presidente Calderón en el Lanzamiento de la Campaña: El agua es como de tu familia, protégela
29/10/09	El Gobierno Federal seguirá con el impulso de las transformaciones de fondo que necesita: Presidente Calderón
30/10/09	El Presidente Calderón en la Ceremonia de la 15ª Entrega de los Premios de Negocios BRAVO

Date	Title
30/10/09	México está más fuerte que nunca: Presidente Calderón
06/11/09	Diversas intervenciones en la Ceremonia de Inauguración del 9° Congreso Mundial de Tierras Silvestres
06/11/09	El Presidente Calderón en la Ceremonia de Inauguración del 9° Congreso Mundial de Tierras Silvestres
06/11/09	Participó el Presidente Calderón en la Ceremonia de Inauguración del 9° Congreso Mundial de Tierras Silvestres en Mérida, Yucatán
14/11/09	Enfatiza el Presidente Calderón la necesidad de asociar los objetivos de reducción de emisiones de carbono con los incentivos económicos
23/11/09	Diversas intervenciones en la Inauguración del Centro Interpretativo El Cielo
23/11/09	El Presidente Calderón en la Inauguración del Centro Interpretativo El Cielo
30/11/09	El Presidente Felipe Calderón en la Sesión de Apertura de la XIX Cumbre Iberoamericana
01/12/09	Participación del Presidente Felipe Calderón en la Segunda Sesión Plenaria de la XIX Cumbre Iberoamericana
04/12/09	El Presidente Felipe Calderón Hinojosa en la Inauguración de la Presa de Almacenamiento “El Gigante”
11/12/09	Diversas intervenciones en el evento de Luz Sustentable
11/12/09	El Presidente Calderón en el evento de Luz Sustentable
14/12/09	Participará el Presidente Calderón en la Conferencia de Cambio Climático (COP-15) en Copenhague, Dinamarca
16/12/09	El Presidente Felipe Calderón en el Mensaje a los Medios de Comunicación que ofreció en el Marco de la 15ª Conferencia de las Naciones Unidas sobre Cambio Climático
16/12/09	Mensaje a Medios en el Marco de la 15ª Conferencia de las Naciones Unidas sobre Cambio Climático
17/12/09	El Presidente Calderón en la Ceremonia de Entrega del Premio Globe
17/12/09	El Presidente Calderón en la Sesión Plenaria de la 15ª Conferencia de las Naciones Unidas sobre Cambio Climático
17/12/09	El Presidente Felipe Calderón se reúne con el Secretario General de la ONU
17/12/09	Exhortó el Presidente Calderón a líderes presentes en Copenhague a demostrar un compromiso con el reto del cambio climático
17/12/09	Ha llegado la hora de pasar de los argumentos a los acuerdos contra el cambio climático, manifiesta el Presidente Calderón
17/12/09	Intercambiaron el Presidente Calderón y el Primer Ministro del Reino Unido, puntos de vista sobre los retos a superar en la Conferencia de Copenhague
17/12/09	Recibió el Presidente Calderón el Premio Globe en la COP-15, al Liderazgo Internacional de Medio Ambiente
18/12/09	El Presidente Calderón participó en la Sesión Plenaria de Adopción del Acuerdo de Copenhague
18/12/09	Mensaje a los Medios de Comunicación del Presidente Felipe Calderón al término de su participación en la 15 Conferencia de las Naciones Unidas sobre Cambio Climático
08/01/10	El Presidente Calderón en la Comida de Trabajo con motivo de la XXI Reunión de Embajadores y Cónsules de México
22/01/10	El Presidente Felipe Calderón en la entrega de cobertores, “Dale Calor a México”
28/01/10	El Presidente Calderón en la Sesión Plenaria titulada: Global Governance Redesigned
29/01/10	Participa el Presidente Calderón en Reunión Informal de Líderes Mundiales, en el Foro Económico de Davos
29/01/10	PARTICIPACIÓN DEL PRESIDENTE CALDERÓN DURANTE LA SESIÓN PLENARIA: FROM COPENHAGEN TO MÉXICO, WHAT´S NEXT?
29/01/10	Sesión Plenaria: From Copenhagen to Mexico, What´s next?
02/02/10	El Presidente Calderón en la Conferencia sobre Cambio Climático COP-16
02/02/10	Segunda intervención del Presidente Calderón en la Conferencia sobre Cambio Climático COP-16
02/02/10	Sesión de Preguntas y Respuestas en la Conferencia: México, una economía emergente. Retos internos y política exterior
21/02/10	Declaración sobre Cambio Climático
21/02/10	Palabras del Presidente Calderón, durante la Ceremonia de Inauguración de la I Cumbre México-CARICOM
23/02/10	Declaración de la Cumbre de la Unidad de América Latina y el Caribe
02/03/10	Palabras del Presidente Felipe Calderón, durante la Entrega de la Obra de Recuperación de Arenales en el Litoral de Benito Juárez y Solidaridad
11/03/10	Diversas intervenciones en el Inicio de Campaña Nacional de Protección contra Incendios Forestales 2010
11/03/10	El Presidente calderón en el Inicio de Campaña Nacional de Protección contra Incendios Forestales 2010
24/03/10	El Presidente Calderón en la Comida de Clausura del XXIV Encuentro Nacional de Vivienda
26/03/10	Diversas intervenciones en la Presentación de la Agenda del Agua 2030
26/03/10	El Presidente Calderón en la Presentación de la Agenda del Agua 2030

Date	Title
30/03/10	El Presidente Calderón en la Inauguración del 12° Foro Internacional de Energía
30/03/10	SIGLO XXI, NUEVA ERA ENERGÉTICA A NIVEL MUNDIAL: FCH
21/04/10	El Presidente Calderón en la Inauguración del Parque Ecológico Jaguaroundi
02/05/10	Intervención del Presidente en la Conferencia Internacional: Construyendo sobre Copenhague para el éxito de México
16/05/10	Se reúne el Presidente Calderón con su homólogo de España
17/05/10	Diversas intervenciones en el Primer Foro del Consejo España-México
18/05/10	Estrechan vínculos México y Finlandia
20/05/10	El Presidente Calderón en su participación en la Sesión Conjunta del Congreso de los Estados Unidos de América
27/05/10	El Presidente Calderón en el Mensaje ante el Pleno del Parlamento
04/06/10	Diversas intervenciones en el Día Mundial del Medio Ambiente 2010
04/06/10	El Presidente Calderón en el Día Mundial del Medio Ambiente 2010
04/06/10	Enfrentar cambio climático demanda acuerdos políticos: FCH
15/06/10	Diversas intervenciones en la Inauguración de la Planta de Tratamiento de Aguas Residuales La Sonoreña
27/06/10	COP-16 en México, parteaguas en la lucha contra cambio climático
01/07/10	El Presidente en la Cena de Estado en honor del Presidente de la República de Corea y de su Esposa
08/07/10	El Presidente en la Reunión de evaluación por los daños del huracán "Alex", Tamaulipas
15/07/10	El Presidente en la Cena Oficial en honor del Primer Ministro de Kuwait Sr. Jeque Nasser Al-Sabah
21/07/10	El Presidente Calderón en el evento sobre el paquete de apoyos para Coahuila, Nuevo León y Tamaulipas
24/07/10	El Presidente en la Conferencia de Jefes de Estado y de Gobierno Africanos sobre Cambio Climático
24/07/10	Llega FCH a Uganda para participar en la Cumbre de la Unión Africana
26/07/10	Palabras de la licenciada Margarita Zavala, presidenta del DIF Nacional y esposa del Presidente de México, durante la premiación del XXXIII Concurso Nacional de Pintura Infantil, "El Niño y la Mar" 2010.
07/08/10	Reunión del Presidente con congresistas de EUA
31/08/10	El Presidente Calderón en la gira de trabajo por el estado de Veracruz
19/09/10	El Presidente Calderón durante la Ceremonia Conmemorativa del Día Nacional de Protección Civil
24/09/10	Segunda intervención del Presidente Calderón en la Reunión de Evaluación por los daños causados por el Huracán Karl en Veracruz
27/09/10	El Presidente Calderón en la Ceremonia Oficial de Bienvenida a México del General Michel Sleiman, Presidente de la República del Líbano
27/09/10	Fortalecen relaciones México y Líbano
29/09/10	El Presidente Calderón en el Foro de Eficiencia Energética y Acceso
29/09/10	Hay cambio climático y afecta a la gente más pobre: FCH
07/10/10	Diversas intervenciones en la Inauguración de la Central Ciclo Combinado Baja California
13/10/10	Diversas intervenciones en Entrega de los Premios Nacionales de Vivienda 2009-2010
13/10/10	El Presidente en Entrega de los Premios Nacionales de Vivienda 2009-2010
26/10/10	El Presidente de Colombia en la Cumbre del Mecanismo de Tuxtla
26/10/10	Llega Presidente Calderón a Colombia
09/11/10	Viaja el Presidente a Corea y Japón
11/11/10	COP16, oportunidad para avanzar hacia un régimen global sobre cambio climático
11/11/10	El Presidente Calderón en el Almuerzo de la Reunión del G20 sobre el Cambio Climático
11/11/10	El Presidente Calderón en la Mesa de Trabajo sobre Crecimiento Verde
11/11/10	Reunión Bilateral con el Primer Ministro de la India
11/11/10	Se reúne el Presidente Calderón con el Primer Ministro de Canadá
12/11/10	Mensaje a medios del Presidente Calderón
12/11/10	Participó el Presidente en la sesión Crecimiento y Medio Ambiente de la Cumbre APEC
12/11/10	Reunión Bilateral con el Primer Ministro de Singapur, Lee Hsien Loong
12/11/10	Sostijan encuentro el Presidente Calderón con el Presidente del Consejo Europeo
13/11/10	El Presidente sostuvo reuniones con Mandatarios de Nueva Zelanda, Vietnam y Australia
17/11/10	Diversas intervenciones en la Inauguración del Centro del Centro de Cultura para la Conservación, Sierra Chincua
24/11/10	El Presidente Calderón en la Ceremonia de Entrega del Premio Nacional de Ciencias y Artes 2010
25/11/10	El Presidente Calderón en el Programa de Prevención y Atención de Desastres Naturales
26/11/10	Diversas intervenciones en la Inauguración de la Planta de Biodiesel
26/11/10	El Presidente Calderón en la Inauguración de la Planta de Biodiesel
26/11/10	La Planta de Biodiesel, compromiso de México en la lucha contra el cambio climático
28/11/10	Diversas intervenciones en la la Inauguración del Aerogenerador Eléctrico Cancún

Date	Title
28/11/10	El Presidente Calderón en la Inauguración del Aerogenerador Eléctrico Cancún
28/11/10	Inaugura el Presidente Felipe Calderón Aerogenerador en Cancún
29/11/10	Diversas intervenciones en la Inauguración de la XVI Conferencia Internacional sobre Cambio Climático, COP16/CMP6
29/11/10	El Presidente Calderón en la Inauguración de la XVI Conferencia Internacional sobre Cambio Climático, COP16/CMP6
29/11/10	El Presidente Felipe Calderón en el Foro Líderes en Acción sobre Cambio Climático 2010
29/11/10	Inauguración de la XVI Conferencia Internacional sobre Cambio Climático
03/12/10	Se reúne el Presidente con el Rey de España, Juan Carlos I
04/12/10	El Presidente Calderón en la Primera Sesión Plenaria de la XX Cumbre Iberoamericana
04/12/10	Reunión del Presidente Calderón con el Presidente de Ecuador, Rafael Correa
04/12/10	Se reúne el Presidente con sus homólogos de Chile, Colombia y Perú
05/12/10	Discurso del Presidente Calderón en el Día Mundial de los Bosques
05/12/10	Diversas intervenciones en el Día Mundial de los Bosques
05/12/10	Diversas Intervenciones en Green Solutions@COP16
05/12/10	El Presidente en Green Solutions@COP16
05/12/10	Green Solutions@COP16, plataforma hacia una economía “verde”
06/12/10	COP16, un éxito al restablecer la confianza en el multilateralismo
06/12/10	Diversas intervenciones en la Estrategias y logros de la vivienda sustentable
06/12/10	El Presidente Calderón en el evento Estrategias y logros de la vivienda sustentable
06/12/10	El Presidente Calderón en el Mensaje a medios sobre el Programa Luz Sustentable y sustitución gradual de focos incandescentes
06/12/10	El Presidente Calderón en Turismo responsable ante el cambio climático: ¿Qué sigue?
06/12/10	El Presidente en la Iniciativa de la Presidenta de la COP16 sobre la Vinculación de Actores Interesados en la Lucha contra el Cambio Climático
06/12/10	Estrategias y logros de la vivienda sustentable
06/12/10	Iniciativa de la Presidenta de la COP16 sobre la Vinculación de Actores Interesados en la Lucha contra el Cambio Climático
06/12/10	Lee el artículo de Octavio Paz citado por el Presidente Calderón
06/12/10	México y Quebec colaboran frente al calentamiento global
06/12/10	Sesión de Preguntas y Respuestas en la Iniciativa de la Presidenta de la COP16 sobre la Vinculación de Actores Interesados en la Lucha contra el Cambio Climático
06/12/10	Turismo responsable ante el cambio climático: ¿Qué sigue?
07/12/10	Conversa Presidente con empresarios asistentes a Green Solutions
07/12/10	Diversas intervenciones en la Inauguración del Segmento de Alto Nivel, COP16
07/12/10	Diversas intervenciones en la Presentación del Documental: De árboles y estrellas
07/12/10	El Presidente Calderón en la Inauguración del Segmento de Alto Nivel, COP16
07/12/10	El Presidente Calderón en la Presentación del Documental: De árboles y estrellas
07/12/10	Participa el Presidente Felipe Calderón Hinojosa en el Encuentro de Alto Nivel sobre Soluciones al Cambio Climático
07/12/10	Se reúne el Presidente Felipe Calderón Hinojosa con el Secretarío General de la ONU, Ban Ki-moon
08/12/10	Diversas intervenciones en el evento de Energética en el Marco Post-2012: Impulsando la oportunidad hacia adelante
08/12/10	El Banco Mundial apoya a México en iniciativas de cambio climático
08/12/10	El Presidente Cálderón en el evento de Energética en el Marco Post-2012: Impulsando la oportunidad hacia adelante
08/12/10	El Presidente Calderón en el evento Trabajo de los Bancos Multilaterales de Desarrollo para un desarrollo sustentable
08/12/10	Encabezará Presidente los Diálogos de alto nivel de la COP16
08/12/10	Evento Energética en el Marco Post-2012
08/12/10	Reunión con la Presidenta de la Confederación Suiza, Doris Leuthard
08/12/10	Se reúne el Presidente Calderón con el Ministro de Petróleo del Reino de Arabia Saudita, Ali Bin Ibrahim Al-naimi
08/12/10	Se reúne el Presidente Calderón con el Primer Ministro de la República de Kenia, Raila Amolo Ondinga
08/12/10	Se reúne el Presidente Calderón con el Primer Ministro de Noruega, Jens Stoltenberg
08/12/10	Visión REDD+, una estrategia para reducir la deforestación
09/12/10	Bolivia expresa importancia del patrimonio indígena y urgencia de acuerdos en COP16
09/12/10	COP16 representa un hito importantísimo para África: Jacob Zuma
09/12/10	El Presidente Calderón en Diálogos de Alto Nivel: Keeping high long-term ambitions against Climate Change
09/12/10	El Presidente Calderón en el Mensaje a medios con el Presidente de Sudáfrica, en el marco de la COP16
09/12/10	El Presidente de Sudáfrica, Jacob Zuma, en el Mensaje a medios, en el marco de la COP16

Date	Title
09/12/10	México agradece a Granada acciones en los preparativos de la COP16
10/12/10	Diversas intervenciones en la Inauguración del Pabellón de Baja Emisión de Co2
10/12/10	El Presidente Calderón en la Inauguración del Pabellón de Baja Emisión de Co2
10/12/10	Inauguración del Pabellón de Baja Emisión de CO2
11/12/10	Acuerdan Fondo Verde en la COP16
11/12/10	Concluye la COP16; Cancún, una acción global compartida: FCH
11/12/10	El Presidente Calderón en la Ceremonia de Clausura de la 16 Conferencia de las Partes de la Convención Marco de las Naciones Unidas sobre cambio climático
11/12/10	Recibe Presidente Calderón felicitación de Barack Obama por la COP16
12/12/10	Dinamarca felicita al Presidente Calderón por el éxito de la COP16
12/12/10	El Secretario General de la ONU felicita al Presidente Calderón por la COP16
13/12/10	COP16 establece las bases para tomar medidas urgentes contra la amenaza global
13/12/10	El Presidente informa sobre logros y resultados acordados en la COP16
13/12/10	Entrevista del Presidente Calderón con Grupo Radio Centro
13/12/10	Mensaje a Medios de Comunicación del Presidente Calderón con motivo de los logros y resultados de la COP16/CMP6
14/12/10	Japón felicita al Presidente Calderón por los resultados de la COP16
15/12/10	Greenpeace reconoce liderazgo del Presidente Calderón en los acuerdos de la COP16
21/01/11	Expresa el Presidente solidaridad al pueblo de Brasil
27/01/11	El Presidente Calderón en Diálogo de Líderes. The Road to Durban: Building on the Cancún Agreements
27/01/11	Reunión Anual del Foro Económico Mundial 2011
28/01/11	Conferencia de Prensa que ofreció el Presidente, en el marco de la Reunión Anual 2011 del Foro Económico Mundial
28/01/11	El Presidente Calderón en Sesión Interactiva: Redefiniendo el Desarrollo Sustentable
28/01/11	Participación del Presidente Felipe Calderón Hinojosa en el Foro: Hacia un nuevo modelo de crecimiento global
28/01/11	Reunión con el Primer Ministro de Dinamarca, Lars Lokke Rasmussen
28/01/11	Reunión con el Primer Ministro de Noruega, Jens Stoltenberg
28/01/11	Reunión con el Primer Ministro de Suecia, Fredrik Reinfeldt
28/01/11	Se reúne el Presidente Calderón con el Presidente de Mitsubishi, Yorihiro Kojima
28/01/11	Sesión Interactiva: Redefiniendo el Desarrollo Sustentable
30/01/11	Participa Presidente Calderón en Diálogo de Líderes en Davos
09/02/11	Diversas intervenciones en el Encuentro con la Academia Nacional de Medicina de México
11/02/11	El Presidente Calderón en la Inauguración de la Planta de Papel SCA
16/02/11	Reunión del Presidente con el Ex Primer Ministro del Reino Unido
07/03/11	Diversas intervenciones en la Comida con motivo de la Toma de Protesta del Consejo Directivo Nacional de CANACINTRA 2011
29/03/11	Diversas Fundación de la Ciudad Rural Sustentable
05/04/11	Diversas intervenciones en la Presentación del Programa de Modernización Sustentable de la Agricultura Tradicional
05/04/11	El Presidente Calderón en la Presentación del Programa de Modernización Sustentable de la Agricultura Tradicional
11/04/11	Diversas intervenciones durante el evento “Cambia tu viejo por uno nuevo”. Entrega del refrigerador un millón
12/04/11	El Presidente Calderón en la Revisión de Operativo de Incendios
12/04/11	Gira de trabajo por el estado de Coahuila
26/04/11	El Presidente Calderón en el Proyecto Estratégico de Biodigestores
10/05/11	Ceremonia de Entrega del Reconocimiento: Champions of the Earth 2011
10/05/11	Conferencia de prensa previa a la Entrega del Reconocimiento: Champions of the Earth
11/05/11	Visita de trabajo a los Estados Unidos de América, Nueva York
17/05/11	El Presidente en la Inauguración de la Planta de Cogeneración de Energía en el Ingenio Tres Valles
20/05/11	Diversas intervenciones durante la Inauguración de la Planta Biogas de Juárez
20/05/11	El Presidente Calderón durante la inauguración de la planta Biogas de Juárez
06/06/11	Día Mundial del Medio Ambiente 2011
06/06/11	El Presidente Calderón en el Día mundial del Medio Ambiente 2011
06/06/11	Pago por Servicios Ambientales
07/06/11	Diversas intervenciones en la Inauguración de la Azotea Verde del Edificio Sede del Instituto del Fondo Nacional de la Vivienda para los Trabajadores
07/06/11	El Presidente Calderón en la Inauguración de la Azotea Verde del Edificio Sede del Instituto del Fondo Nacional de la Vivienda para los Trabajadores
12/06/11	El Presidente Calderón en la Ceremonia de Graduación de estudiantes de Stanford
14/06/11	Diversas intervenciones durante los festejos del Día Mundial del Medio Ambiente
21/06/11	El Presidente Calderón en el Lanzamiento del Progama Mundo Maya

Date	Title
05/07/11	El Presidente Calderón en el Lanzamiento del Programa de Luz Sustentable
05/07/11	Gira de trabajo por Morelos
07/07/11	Diversas intervenciones en el anuncio de inversión de México Power Group 2011
17/07/11	El Presidente Calderón en la Jornada Nacional de Reforestación 2011 – Guanajuato
17/07/11	El Presidente Calderón en la Jornada Nacional de Reforestación 2011 – Tlaxcala
19/07/11	El Presidente Calderón en la Puesta en marcha de la segunda etapa del programa Agua para siempre
03/08/11	Diversas intervenciones en la ceremonia del 60º Aniversario de DINA
12/09/11	El Presidente Calderón en el Mensaje con motivo del Quinto Informe de Gobierno Región Centro Sur
19/09/11	Inicia el Presidente Visita de Trabajo en Nueva York y Los Ángeles
20/09/11	El Presidente Calderón en la sesión inaugural de la Clinton Global Initiative 2011
20/09/11	Visita de trabajo del Presidente Calderón por EUA
21/09/11	Ahorro sustentable para las familias
21/09/11	El Presidente Calderón en el Debate General de la 66° Sesión de la Asamblea General de las Naciones Unidas
21/09/11	El Presidente Calderón en la Asamblea General de la ONU
22/09/11	El Presidente Calderón en la Inauguración de la 9ª Expo Forestal, México Siglo XXI
03/10/11	Día Mundial del Hábitat
03/10/11	Diversas intervenciones en el Día Mundial del Hábitat 2011
03/10/11	El Presidente Calderón en el Día Mundial del Hábitat 2011
21/10/11	Diversas intervenciones durante el Programa Luz Sustentable y el lanzamiento del Programa de Luz Eficiente
21/10/11	El Presidente Calderón durante el Programa Luz Sustentable y el lanzamiento del Programa de Luz Eficiente
21/10/11	Entrega de la lámpara ahorradora 6 millones
03/11/11	10 grandes transformaciones de México: Política Exterior
03/11/11	Palabras del Presidente Calderón durante la Sesión Plenaria del B-20: Crecimiento Verde
04/11/11	Mensaje del Presidente Calderón en el marco de actividades de la Cumbre de Líderes del G-20
08/11/11	Diversas intervenciones en el Anuncio del cambio estructural en el mercado de gas natural
24/11/11	Diversas intervenciones en el evento Green Solutions 2011
24/11/11	El Presidente Calderón en el evento Green Solutions 2011
24/11/11	Green Solutions 2011
01/12/11	10 grandes transformaciones de México: Cuidado del medio ambiente
01/12/11	Apoyo a estados afectados por sequías
01/12/11	Diversas intervenciones en la Estrategia de atención a las afectaciones por sequía
01/12/11	El Presidente Calderón en la Estrategia de atención a las afectaciones por sequía
04/12/11	El Presidente Calderón en su mensaje por el Quinto Año de Gobierno
13/12/11	El Presidente Calderón en el Seminario G20 en México: Los retos actuales para el crecimiento económico mundial
13/12/11	Seminario del G20: Los retos actuales para el crecimiento económico global
14/12/11	Diversas intervenciones en la Ceremonia de Entrega del Premio de Ecología y Medio Ambiente Miguel Alemán Valdés 2011
14/12/11	El Presidente Calderón en la Ceremonia de Entrega del Premio de Ecología y Medio Ambiente Miguel Alemán Valdés 2011
05/01/12	Canciller Patricia Espinosa-XXIII Reunión Anual de Embajadores y Cónsules de México
19/01/12	Diversas intervenciones en el Programa Multianual de Cambio Climático Global 2012-2016
19/01/12	El Presidente Calderón en el Programa Multianual de Cambio Climático Global 2012-2016
19/01/12	México enfrenta al cambio climático
26/01/12	Sesión pública Stand Alone, Global Economic Crisis: Role and Challenges of the G-20
27/01/12	Alianza Mexicana de Agronegocios para el Crecimiento Sustentable
03/02/12	El Presidente Calderón en la Reunión con los Sherpas del G-20 y representantes de organismos internacionales
15/02/12	Ahorro de energía, ahorro de dinero
15/02/12	Diversas intervenciones en la Entrega del electrodoméstico millón y medio y de 17 millones de lámparas, en los programas Sustitución de Electrodomésticos y Luz Sustentable
15/02/12	El Presidente Calderón en la Entrega del electrodoméstico millón y medio y de 17 millones de lámparas, en los programas Sustitución de Electrodomésticos y Luz Sustentable
20/02/12	Mensaje al Grupo del G-20
21/02/12	Diversas intervenciones en la Supervisión de la Planta de Tratamiento de Aguas Residuales de Atotonilco
03/03/12	El Presidente Calderón durante el evento Meta cumplida: 6 millones de financiamientos para vivienda
07/03/12	Diversas intervenciones en la Inauguración de las Centrales Eólicas Oaxaca II, III y IV
07/03/12	El Presidente Calderón en la Inauguración de las Centrales Eólicas Oaxaca II, III y IV

Date	Title
11/03/12	Diversas intervenciones en la Supervisión de avances de la Central Hidroeléctrica La Yesca
11/03/12	El Presidente Calderón en la Supervisión de avances de la Central Hidroeléctrica La Yesca
12/03/12	El Presidente Calderón en la Inauguración del cauce del alivio Sabanilla
15/03/12	20 Aniversario de la Conabio
15/03/12	El Presidente Calderón durante el Anuncio del Programa de Saneamiento de la Cuenca Alta del Río Amecameca
15/03/12	El Presidente Calderón en la Celebración del XX Aniversario de la Comisión Nacional para el Conocimiento y Uso de la Biodiversidad
16/03/12	El Presidente Calderón en la Inauguración de la planta de tratamiento de aguas residuales de Acapantzingo
17/03/12	Agua limpia para México
17/03/12	Centro Nacional de Recursos Genéticos Proyecto Bicentenario
17/03/12	Diversas intervenciones en la Inauguración del Centro Nacional de Recursos Genéticos
17/03/12	El Presidente Calderón en la Inauguración de la Planta de Tratamiento de Aguas Residuales: El Ahogado
17/03/12	El Presidente Calderón en la Inauguración del Centro Nacional de Recursos Genéticos
18/03/12	Diversas intervenciones en el 74 Aniversario de la Expropiación Petrolera y entrega de obra de la Planta Reformadora NAFTA
18/03/12	El Presidente Calderón en el 74 Aniversario de la Expropiación Petrolera y entrega de obra de la Planta Reformadora NAFTA
21/03/12	Diversas intervenciones en la Entrega de vivienda, obras y acciones para la recuperación de Angangueo
23/03/12	Centro Desarrollo Sustentable
23/03/12	Diversas intervenciones en la Inauguración del Centro “Desarrollo Sustentable, A.C.”
23/03/12	El Presidente Calderón en la Inauguración del Centro “Desarrollo Sustentable, A.C.”
26/03/12	El Presidente Calderón en la Comida de bienvenida con representantes de la Industria Turística Nacional e Internacional
28/03/12	El Presidente Felipe Calderón en el evento: Un Gobierno Democrático que rinde cuentas
28/03/12	Gobierno democrático que rinde cuentas
02/04/12	Declaración conjunta de los Líderes de América del Norte
04/05/12	Diversas intervenciones en el Recorrido por el Paseo Río Atoyac y el Ecoparque Metropolitano Puebla
04/05/12	El Presidente Calderón en el Recorrido por el Paseo Río Atoyac y el Ecoparque Metropolitano Puebla
09/05/12	El Presidente Calderón en la Reunión de evaluación por la sequía en Sonora
18/05/12	El Presidente Calderón en la Reunión de Ministros de trabajo y empleo del G20
21/05/12	El Presidente Felipe Calderón durante el mensaje a medios de comunicación de la II Cumbre México-Caricom
05/06/12	Decreto de la Ley General de Cambio Climático
05/06/12	El Presidente Calderón en su Mensaje con motivo del Día Mundial del Medio Ambiente
05/06/12	G20 Young Entrepreneur Summit
12/06/12	El Presidente Calderón en su Mensaje a medios con motivo de la Cumbre de Líderes del G20
12/06/12	México, anfitrión de la Cumbre de Líderes del G20
15/06/12	VII Cumbre de Líderes del G20
16/06/12	El Presidente Calderón en la Ceremonia de imposición de la condecoración de la Orden Mexicana del Águila Azteca
16/06/12	El Presidente Calderón en la Inauguración del Centro de Convenciones
16/06/12	Imposición de la Orden Mexicana del Águila Azteca
17/06/12	Diversas intervenciones durante la Ceremonia de Imposición de la Condecoración de la Orden Mexicana del Águila Azteca
17/06/12	Se reúne el Presidente Calderón con el Presidente de la República de Corea, Lee Myung-bak
18/06/12	Se reúne el Presidente Calderón con la Canciller Federal de Alemania, Angela Merkel
19/06/12	El Presidente Calderón en la Conferencia de Prensa ofrecida a medios de comunicación
26/07/12	Diversas intervenciones en el Lanzamiento de la segunda etapa del Programa Luz Sustentable
26/07/12	El Presidente Calderón en el Lanzamiento de la segunda etapa del Programa Luz Sustentable
14/08/12	Diversas intervenciones en la Conmemoración del 75 aniversario de la Comisión Federal de Electricidad
28/08/12	Reunión con el Presidente del Panel Intergubernamental de Cambio Climático
03/09/12	El Presidente Calderón durante su Mensaje con motivo del Sexto Informe de Gobierno
24/09/12	El Presidente Calderón en la Premiere global del Vuelo de la Monarca
26/09/12	El Presidente Calderón en la Participación en el Debate General de la 67° Sesión de la Asamblea General de las Naciones Unidas
04/10/12	Diversas intervenciones durante la inauguración de la Presa de Almacenamiento Vista Hermosa
09/10/12	Diversas intervenciones en la Inauguración de la Exhibición Green Solutions 2012
09/10/12	El Presidente Calderón en la Inauguración de la Exhibición Green Solutions 2012

Date	Title
09/10/12	Green Solutions 2012
12/10/12	Desarrollo Sustentable en BCS
12/10/12	Diversas intervenciones en la Inauguración de la Central Solar Fotovoltaica
12/10/12	El Presidente Calderón en la Inauguración de la Central Solar Fotovoltaica
15/10/12	El Presidente Felipe Calderón en la Inauguración de la Planta de Tratamiento de Aguas Residuales de la Residencia Oficial de Los Pinos
15/10/12	Los Pinos reduce su huella ambiental
25/10/12	Diversas intervenciones en la Inauguración de la Planta de Cogeneración Nuevo Pemex
25/10/12	El Presidente Calderón en la Inauguración de la Planta de Cogeneración Nuevo Pemex
25/10/12	El Presidente Calderón en la Inauguración de planta extractora de aceite de palma
25/10/12	Gira de trabajo por el estado de Tabasco
30/10/12	Diversas intervenciones en la Inauguración de las Centrales Eólicas Oaxaca I y La Venta III
30/10/12	Diversas intervenciones en la Inauguración del Parque Eólico Piedra Larga de Grupo Bimbo
30/10/12	El Presidente Calderón en la Inauguración de la Línea 12, Línea Dorada del Sistema de Transporte Colectivo Metro
30/10/12	El Presidente Calderón en la Inauguración de las Centrales Eólicas Oaxaca I y La Venta III
30/10/12	El Presidente Calderón en la Inauguración del Parque Eólico Piedra Larga de Grupo Bimbo
30/10/12	Se inaugura la Línea Dorada del Metro
31/10/12	El Presidente Calderón en la Inauguración del Centro Hidrometeorológico Regional: Tuxtla Gutiérrez
06/11/12	El Presidente Felipe Calderón durante la Inauguración de la Central Hidroeléctrica: La Yesca
12/11/12	Diversas intervenciones en la Ceremonia de abanderamiento de los Buques Tanque: Miguel Hidalgo II y Vicente Guerrero II y Anuncio del Inicio de los Gasoductos del Sistema Integral Norte-Noroeste
17/11/12	El Presidente Felipe Calderón durante la I Sesión Plenaria
25/11/12	Diversas intervenciones durante la Inauguración de la Planta Criogénica de Poza Rica
29/11/12	El Presidente Calderón en la Inauguración del Parque Sustentable Bioparque Urbano San Antonio
29/01/13	Diversas Intervenciones Durante la Instalacion de la Comision Intersecretarial de Cambio Climatico
29/01/13	El Gobierno de la Republica decidido a impulsar para México un crecimiento de beneficio social
29/01/13	Instalación de la Comisión Intersecretarial de Cambio Climático
29/01/13	Sistema Nacional de Cambio Climático
31/01/13	Desarrollo Sustentable de Bosques
27/02/13	Comisión Intersecretarial de Cambio Climático inicia estrategia nacional
22/03/13	Diversas intervenciones durante el Día Mundial del Agua
09/04/13	El Presidente Enrique Peña Nieto delineó la Política Exterior que México seguirá en los próximos años
09/04/13	Conclusión de la gira de trabajo por la República Popular China y Japón
03/06/13	Impulsa el Presidente Peña Nieto una política ambiental para asegurar una mejor calidad de vida para los mexicanos
03/06/13	Diversas intervenciones durante la Semana Nacional del Medio Ambiente
03/06/13	Estrategia Nacional de Cambio Climático
03/06/13	Semana del Medio Ambiente
03/06/13	Semana Nacional del Medio Ambiente
07/06/13	Mil 800 mdp al sector ganadero afectado por efectos del cambio climático
23/08/13	Diversas intervenciones durante la Firma del Convenio de la Comisión Ambiental de la Megalópolis
23/08/13	Expresa EPN su reconocimiento a diputados y senadores por los avances legislativos alcanzados en las recientes sesiones extraordinarias
02/09/13	Mensaje del Presidente de los Estados Unidos Mexicanos, licenciado Enrique Peña Nieto, con motivo de su Primer Informe de Gobierno
08/09/13	Presentación de la Iniciativa de Reforma Hacendaria, que tuvo lugar en el Salón Adolfo López Mateos de la Residencia Oficial.
27/09/13	Palabras de Fredrik Reinfeldt, Primer Ministro de Suecia, durante la Comida que ofreció en su Honor el Presidente Enrique Peña Nieto
27/09/13	Se refuerza el diálogo político y se impulsa la relación entre México y Suecia
28/09/13	Los eventos climáticos no van a frenar el desarrollo del país: Enrique Peña Nieto
16/10/13	Acuerdan México y Portugal el fortalecimiento de su relación bilateral y multilateral
21/10/13	Palabras del señor Michael D. Higgins, Presidente de Irlanda, durante la comida que ofreció en su honor el Presidente Enrique Peña Nieto
16/12/13	Relaciones diplomáticas México Turquía
17/12/13	Marco de cooperación estratégica y asociación México-Turquía para el Siglo XXI
20/12/13	Palabras del Presidente Enrique Peña Nieto, durante la Promulgación de la Reforma Constitucional en Materia Energética

Date	Title
20/01/14	Palabras, Presidente Enrique Peña Nieto, Reunión Cruz Roja Mexicana, a Beneficio de los Damnificados de los Huracanes "Ingrid" y "Manuel"
26/03/14	El objetivo de la Reforma Energética es generar energía más limpia y más barata para hacer de México un país más competitivo: EPN
26/04/14	Inauguración de la Central Fotovoltaica Aura Solar I
28/04/14	Presenta el Presidente Peña Nieto el Programa Nacional de Infraestructura 2014 -2018; prevé una inversión global de 7.7 billones de pesos
30/04/14	Declaración De Mérida
28/05/14	¿Qué es el Fondo para el Medio Ambiente Mundial?
28/05/14	Aportará México 20 mdd al Fondo para el Medio Ambiente Mundial en el periodo 2014-2018: Enrique Peña Nieto
28/05/14	Beneficios Ambientales de la Reforma Energética
28/05/14	Palabras del Presidente Enrique Peña Nieto, durante la Quinta Asamblea del Fondo para el Medio Ambiente Mundial
08/06/14	El Gobierno de la República sustituirá 32 millones de lámparas incandescentes por ahorradoras de energía
20/06/14	Mensaje conjunto a medios de comunicación que ofrecieron los Presidentes Enrique Peña Nieto; Michelle Bachelet; Juan Manuel Santos, y Ol
26/06/14	Palabras del Presidente Enrique Peña Nieto, durante la Reunión con Gobernadores del Consejo Mundial del Agua
10/07/14	Día del Árbol en México
17/07/14	Palabras del señor Ollanta Humala, Presidente de Perú, durante la comida que ofreció en su honor el Presidente Enrique Peña Nieto
17/07/14	Visita Oficial del Presidente de la República del Perú, señor Ollanta Humala Tasso, a los Estados Unidos Mexicanos: Comunicado conjunto
25/07/14	La relación entre México y Japón es más productiva y confianza; el comercio entre ambos países es cercano a 20 mil millones de dólares: EPN
25/07/14	Mensaje a medios de comunicación del señor Shinzo Abe, Primer Ministro de Japón, en el marco de su Visita Oficial
25/07/14	Visita Oficial del Primer Ministro del Japón, Shinzo Abe. Comunicado conjunto
21/09/14	El Presidente Enrique Peña Nieto se reunió con el Secretario General de la ONU, Ban Ki-Moon
22/09/14	Participación del Presidente Peña Nieto en la AGONU
23/09/14	Frente al Cambio Climático, todos podemos ser la solución
23/09/14	Intervenciones de Presidente Enrique Peña Nieto en su participación como Copresidente de Mesa sobre Financiamiento de Cumbre del Clima 2014
23/09/14	Palabras del Presidente Enrique Peña Nieto, durante la Sesión Plenaria de Jefes de Estado y/o Gobierno de la Cumbre sobre el Clima 2014
23/09/14	Para México, enfrentar el cambio climático es un compromiso de Estado: Presidente Enrique Peña Nieto
16/10/14	México será sede de la Conferencia de la Diversidad Biológica en 2016, anuncia el Presidente Enrique Peña Nieto
16/10/14	Palabras del Presidente Enrique Peña Nieto, durante la Inauguración del Foro Internacional Alternativas Verdes
03/11/14	Diversas intervenciones durante la Expedición de los Reglamentos de la Reforma Energética
10/12/14	Acción colectiva frente al Cambio Climático
10/12/14	Conferencia de las Partes #COP20
10/12/14	Convención Marco de la ONU sobre el Cambio Climático
10/12/14	El compromiso de México para enfrentar el cambio climático es firme y creciente: Enrique Peña Nieto
10/12/14	El Presidente Enrique Peña Nieto se reunirá con mandatarios de la Alianza del Pacífico
10/12/14	Palabras del Presidente Enrique Peña Nieto durante la Presentación de Declaración de la Alianza del Pacífico en materia de Cambio Climático
12/02/15	Palabras de Le Linh Lan, Embajadora de Vietnam, durante la Presentación de sus Cartas Credenciales al Presidente Enrique Peña Nieto
24/02/15	Diversas intervenciones durante la 48 Reunión Ordinaria de la Conferencia Nacional de Gobernadores
04/03/15	En materia energética estamos impulsado un modelo ganador en beneficio de la sociedad mexicana: Enrique Peña Nieto
04/03/15	La Reforma Energética cuida el Medio Ambiente
04/03/15	Palabras del Presidente Enrique Peña Nieto, durante la Firma de instrumentos en materia de Energía, Cadenas Productivas y Cambio Climático
23/03/15	Diversas intervenciones durante la Entrega de la Planta Tratadora de Aguas Residuales Tuchtlán
26/03/15	Declaración Conjunta sobre Cooperación en Política Ambiental México - Estados Unidos
09/04/15	México se ofrece como mediador para consolidar diálogo EE. UU. – Cuba

Date	Title
12/04/15	El Gobierno de la República está comprometido en transformar a México en una sociedad del conocimiento: Enrique Peña Nieto
21/04/15	Día Internacional de la Madre Tierra 2015
21/04/15	México comprometido con el combate al cambio climático
07/05/15	Visita de Estado a México del Presidente de Colombia, Juan Manuel Santos Calderón
13/05/15	Ratifica el Presidente EPN el compromiso del Gobierno de la República de salvaguardar la vida y el patrimonio de los mexicanos
27/05/15	Hacia una Carta Internacional de Datos Abiertos
09/06/15	La participación de México en la Cumbre EU - CELAC contribuirá a reforzar vínculos ante los desafíos del mundo actual: Enrique Peña Nieto
10/06/15	Relación bilateral México - Unión Europea
02/07/15	México forma parte de la Alianza del Pacífico
10/07/15	Diversas intervenciones en el inicio de la Campaña de Reforestación 2015, Día del Árbol, que tuvo lugar en Lerma, Estado de México
10/07/15	Reafirma el Presidente Enrique Peña Nieto el compromiso del Gobierno de la República de preservar el Patrimonio Natural de México
15/07/15	México y Francia tenemos un compromiso irrenunciable con el medio ambiente: Enrique Peña Nieto
15/07/15	México y Francia tienen un compromiso con el medio ambiente
16/07/15	Firmaron México y Francia más de 60 acuerdos de cooperación en distintos ámbitos, en el marco de una visita de estado histórica: EPN
11/08/15	Diversas intervenciones durante el anuncio del Plan de Negocios de Cargill México 2015-2018
13/08/15	Firman México y Chile acuerdos que darán un nuevo impulso a la relación bilateral
02/09/15	Mensaje del Licenciado Enrique Peña Nieto, Presidente de los Estados Unidos Mexicanos
02/09/15	Mensaje del Presidente Enrique Peña Nieto
03/09/15	Entrevista por Adela. Enrique Peña Nieto: Admito que México enfrenta una situación de desconfianza
15/09/15	Expresa el Presidente Peña Nieto a su homólogo egipcio el dolor y la indignación de la sociedad mexicana ante los sucesos del domingo pasado
18/09/15	Diversas intervenciones durante la Ceremonia de Entrega del Premio Nacional de Protección Civil 2015
24/09/15	Agenda en 70 Asamblea de la ONU
24/09/15	Asistirá el Presidente Peña Nieto a la 70º Asamblea General de la ONU
24/09/15	México asume sus responsabilidades como actor global y confirma convicción de que se fortalece con diálogo y esfuerzo común: Eduardo Sánchez
25/09/15	¿Sabías que en Michoacán el 100% de la energía eléctrica se genera a partir de fuentes renovables?
25/09/15	Central Geo Termo Eléctrica Los Azufres III, Fase I
27/09/15	Cumbre de Adopción de la Agenda 2030
27/09/15	Cumbre sobre el Desarrollo Sostenible 2015
27/09/15	Participación del Lic. Enrique Peña Nieto en la comida privada de Jefes de Estado y Gobierno sobre Cambio Climático
28/09/15	Alianza de Datos para el Desarrollo Sostenible
28/09/15	Intervención del Presidente Enrique Peña Nieto, en el Debate General de la 70ª Asamblea General de la Organización de las Naciones Unidas
28/09/15	Retos ante el cambio climatico y la migracion
29/09/15	Acciones concretas por el Desarrollo Sostenible
12/10/15	Reunión con el Primer Ministro de Quebec
12/10/15	Se reúne el Presidente Enrique Peña Nieto con el Primer Ministro de Quebec
19/10/15	El Presidente Enrique Peña Nieto se suma a la iniciativa mundial para reducir las emisiones de bióxido de carbono
20/10/15	Diversas Encuentro Ministros de Agricultura de las Américas 2015 y 18ª Reunión Ordinaria Junta Interamericana Agricultura del Instituto IICA
20/10/15	La productividad es la mejor vía para conducir al campo de las Américas hacia un mejor destino: EPN
20/10/15	México se suma a la iniciativa mundial para reducir las emisiones de bióxido de carbono
20/10/15	Palabras del Presidente EPN, Inauguración Encuentro de Ministros de Agricultura de las Américas 2015 y 18ª Reunión Ordinaria del IICA
25/10/15	¿Cuánta energía eléctrica se ahorró con el Horario de Verano?
26/10/15	Vaquita Marina: riqueza natural endémica de nuestro país
13/11/15	¡Desarrolla una app web! Participa en el Reto de Cambio Climático
13/11/15	Palabras del Presidente, licenciado Enrique Peña Nieto, durante la Sesión Ordinaria del Consejo Nacional de Protección Civil
15/11/15	El Presidente Enrique Peña Nieto se reunió con el Primer Ministro de Canadá, Justin Trudeau
15/11/15	El Presidente Peña Nieto se reúne con el Primer Ministro de Canadá

Date	Title
16/11/15	Las Naciones del G20 deben facilitar e incrementar su comercio para lograr un crecimiento economico sostenido e incluyente : EPN
16/11/15	México es la 5ª economía más abierta del G20
19/11/15	Los líderes de la APEC tenemos la responsabilidad de crear condiciones para alcanzar crecimientos elevados, sostenibles e incluyentes: EPN
19/11/15	México no está aislado del mundo; es un actor global que participa en distintos foros mundiales: Enrique Peña Nieto
26/11/15	Asistirá el Presidente Enrique Peña Nieto a la COP21, en París, Francia, para hacer frente al cambio climático
26/11/15	El Presidente Peña Nieto asistirá a la COP21 para hacer frente al cambio climático
27/11/15	Los bosques ayudan a reducir los efectos del cambio climático
29/11/15	Conferencia de las Naciones Unidas sobre el Cambio Climático-COP 21
29/11/15	Conferencia de las Partes COP21 sobre cambio climatico París, Francia
29/11/15	Convencion Marco de la ONU sobre el Cambio Climatico
30/11/15	En la COP21 decidimos en qué mundo queremos vivir y qué calidad de vida queremos para el Siglo 21: Enrique Peña Nieto
30/11/15	Fijar un precio al carbono permite pasar de tecnologías contaminantes a energías más limpias y sustentables: Enrique Peña Nieto
30/11/15	México respalda la iniciativa de poner un precio al carbono
30/11/15	Participación del Presidente de los Estados Unidos Mexicanos, Enrique Peña Nieto, durante la presentación de la Iniciativa Carbon Pricing
01/12/15	El Gobierno de la República seguirá trabajando con toda la sociedad para que México desarrolle su enorme potencial: Enrique Peña Nieto
03/12/15	Diversas intervenciones durante la Inauguración del Parque Científico Tecnológico de Yucatán
08/12/15	Anuncia el Presidente Enrique Peña Nieto inversiones verdes de Pemex por 23 mil millones de dólares
09/12/15	Diversas intervenciones durante la Inauguración de la Fábrica Nestlé Purina
17/12/15	Diversas intervenciones durante la 32 Asamblea General Ordinaria del Consejo Nacional Agropecuario
15/01/16	Con la Reforma Energética, la energía eléctrica en México será de mayor calidad, más eficiente y, sobre todo, más barata y más limpia: EPN
15/01/16	Diversas intervenciones durante la Inauguración de la Fábrica de Paneles Solares IUSASOL y la Central Solar Fotovoltaica "Don Alejo"
17/01/16	Palabras del Presidente de los Estados Unidos Mexicanos, licenciado Enrique Peña Nieto, durante el evento Reforma Energética de México
18/01/16	México impulsa una audaz transición energética hacia fuentes más limpias y renovables: Enrique Peña Nieto
18/01/16	Palabras del Presidente Enrique Peña Nieto, durante su Participación en la inauguración de la Cumbre Mundial de la Energía del Futuro 2016
19/01/16	Acuerdos de cooperación bilateral México-Emiratos Árabes Unidos
19/01/16	México, como país abierto al mundo, busca la interconexión con otras naciones para alcanzar mayor desarrollo: Enrique Peña Nieto
22/01/16	Invita el Secretario General de la ONU al Presidente Enrique Peña Nieto a Copresidir el Grupo de Alto Nivel sobre el Agua
05/02/16	Diversas, Ceremonia del 99 Aniversario de la Promulgación de la Constitución Política de los Estados Unidos Mexicanos de 1917
26/02/16	Mensaje a medios de comunicación: Juan Orlando Hernández Alvarado, Presidente de la República de Honduras, en su Visita Oficial a México
29/02/16	Diversas intervenciones durante la 50 Reunión Ordinaria de la Conferencia Nacional de Gobernadores
29/02/16	Llama el Presidente Enrique Peña Nieto al Legislativo a debatir la iniciativa para el Mando Único en las entidades federativas del país
01/03/16	Diversas intervenciones durante la 34ª Conferencia Regional de la FAO para América Latina y el Caribe
01/03/16	En 2015 las exportaciones agroalimentarias ascendieron a 26,373 MDD; superaron en 16.6% a las registradas al inicio de la administración:EPN
03/03/16	Diversas intervenciones durante la Inauguración de la Central Eólica Sureste I, Fase II
22/03/16	Equipamiento de pozos agrícolas y modernización de Unidades y Distritos de Riego
22/03/16	Palabras Presidente Enrique Peña Nieto, Entrega de equipamiento de Pozos Agrícolas y de la modernización de Unidades y Distritos de Riego
07/04/16	Diversas intervenciones durante la conmemoración del Día Mundial de la Salud
11/04/16	La Visita de Estado a la República Federal de Alemania confirman el dinamismo, la dimensión y la profundidad de la relación bilateral: EPN
11/04/16	Palabras del Presidente Enrique Peña Nieto, Cena de Estado que le ofreció el Presidente de la República Federal de Alemania, Joachim Gauck

Date	Title
11/04/16	Palabras Presidente República Federal de Alemania, Joachim Gauck, Cena de Estado que ofreció en honor del Presidente Enrique Peña Nieto
13/04/16	Construyendo una nueva etapa de amistad y colaboración
20/04/16	Mensaje a medios del Presidente del Consejo de Ministros de la República Italiana, Matteo Renzi, en el marco de su Visita Oficial
20/04/16	Recibe el Presidente Enrique Peña Nieto al Presidente del Consejo de Ministros de la República Italiana
04/05/16	El Presidente Enrique Peña Nieto asistirá a la Cumbre de Líderes de América del Norte
06/05/16	Diversas, evento 30 Años del Sistema Nacional de Protección Civil e Inicio de la Temporada de Lluvias y Ciclones Tropicales 2016
06/05/16	Palabras del Presidente EPN, evento: 30 Años del Sistema Nacional de Protección Civil e Inicio Temporada Lluvias y Ciclones Tropicales 2016
24/05/16	Diversas intervenciones durante la Inauguración del 36 Periodo de Sesiones de la Comisión Económica para América Latina y el Caribe
24/05/16	Palabras Presidente de los Estados Unidos Mexicanos, Enrique Peña Nieto, durante la Inauguración del 36 Periodo de Sesiones de la CEPAL
24/05/16	Ratifica el Presidente Enrique Peña Nieto el compromiso de México con la integración y el desarrollo de América Latina y el Caribe
08/06/16	Acuerdan México y la India escalar su relación para pasar a una asociación estratégica
08/06/16	Mensaje que ofreció el Presidente EPN, en el marco de la Visita de Trabajo del Primer Ministro de la República de la India, Narendra Modi
09/06/16	Declaración Conjunta México-India con motivo de la Visita de Trabajo a México del Primer Ministro de la India, Narendra Modi
10/06/16	Declaración Conjunta entre los Estados Unidos Mexicanos y la República de Singapur
22/06/16	México avanza por la ruta de la responsabilidad global
26/06/16	Es el único foro periódico de encuentro trilateral al más alto nivel. Participarán: Enrique Peña Nieto por México, Barack Obama por los Estados Unidos y el anfitrión, Justin Trudeau, por Canadá.
27/06/16	Canadá y México Trabajando por la prosperidad de América del Norte
27/06/16	El respeto a la diversidad y la inclusión social, valores compartidos por Canadá y México: Enrique Peña Nieto
27/06/16	Mensaje a medios de comunicación del Presidente de los Estados Unidos Mexicanos, Enrique Peña Nieto, en el marco de su Visita de Estado
27/06/16	Palabras del Presidente Enrique Peña Nieto, durante la cena que ofreció en su honor el Primer Ministro de Canadá, Justin Trudeau
27/06/16	Palabras del Presidente Enrique Peña Nieto, en la comida que sostuvo con integrantes de la Cámara de Comercio e Industria de Quebec
27/06/16	Palabras del Primer Ministro de Canadá, Justin Trudeau, en la cena que ofreció en honor del Presidente EPN, en el marco de Visita de Estado
27/06/16	Visita de Estado a Canadá: Acuerdos de cooperación con Quebec
28/06/16	Declaración conjunta del Presidente de los Estados Unidos Mexicanos y el Primer Ministro de Canadá
28/06/16	Mensaje a medios de comunicación del Primer Ministro de Canadá, Justin Trudeau, en la Visita de Estado del Presidente Enrique Peña Nieto
29/06/16	#CLAN2016 Cambio Climático
29/06/16	#CLAN2016 Energía Limpia y Segura
29/06/16	#CLAN2016 Frente común ante Cambio Climático
29/06/16	#CLAN2016 Reducción de Contaminantes Climáticos de Vida Corta
29/06/16	#CLAN2016 Transporte Limpio y Eficiente
29/06/16	#CLAN2016 Canadá, EU y México, encuentro trilateral al más alto nivel
29/06/16	Declaración de Líderes de América del Norte sobre la Alianza del clima, energía limpia y medio ambiente
29/06/16	Frente a los retos globales, el aislacionismo no es la solución: Enrique Peña Nieto
29/06/16	Mensaje a medios de comunicación que ofreció el Presidente Enrique Peña Nieto, en el marco de la Cumbre de Líderes de América del Norte
29/06/16	Mensaje a medios del Presidente Enrique Peña Nieto, al término de la reunión bilateral con el Presidente de los Estados Unidos, Barack Obama
29/06/16	Palabras del Presidente de EU, Barack Obama, después de reunión bilateral con Presidente de los Estados Unidos Mexicanos, Enrique Peña Nieto
29/06/16	Para generar prosperidad, el aislamiento no es ruta; la integración lo es: Enrique Peña Nieto
29/06/16	Reunión bilateral México-Estados Unidos
30/06/16	#CLAN2016 Plan de Acción de América del Norte sobre la Alianza del Clima, Energía Limpia y Medio Ambiente
30/06/16	#CLAN2016 Prosperidad económica, comercio y competitividad
30/06/16	#CLAN2016 Temas regionales

Date	Title
30/06/16	Las claves de la XI Cumbre de la Alianza del Pacífico
04/07/16	Mensaje a medios de comunicación del Presidente Enrique Peña Nieto, en la Visita de Estado del Presidente de la República Italiana
04/07/16	México e Italia buscan fortalecer y ampliar la relación bilateral en material comercial, de cooperación y de inversiones recíprocas: EPN
14/07/16	Diversas intervenciones durante el Inicio de la Campaña Nacional de Reforestación 2016 y Celebración del Día del Árbol
14/07/16	El Gobierno de la República se ha trazado el objetivo de reforestar un millón de hectáreas en esta administración: Enrique Peña Nieto
21/07/16	La relación con Estados Unidos significa más productividad, comercio, intercambios y mayor prosperidad para los mexicanos: Eduardo Sánchez
21/07/16	Mensaje a medios: Secretaria de Relaciones Exteriores, Claudia Ruiz Massieu, y el Vocero del Gobierno de la República, Eduardo Sánchez
21/07/16	México y Estados Unidos además de vecinos, son aliados estratégicos
21/07/16	Relación México - Estados Unidos
22/07/16	El Gobierno de México observará con gran interés el proceso electoral de los Estados Unidos, pero no opinará ni se involucrará: EPN
22/07/16	Mensaje a medios de comunicación del Presidente Enrique Peña Nieto, durante su Visita Oficial a los Estados Unidos de América.
08/08/16	Diversas intervenciones durante la Sesión del Sistema Nacional para la Igualdad entre Mujeres y Hombres
26/08/16	Declaración Conjunta México - Paraguay
05/09/16	Mensaje a medios de comunicación que ofreció el Presidente de la República, Enrique Peña Nieto, al finalizar la Cumbre de Líderes del G20
07/09/16	Diversas intervenciones durante la Entrega del Ecoparque Centenario "Toma de Zacatecas" y Casas del Programa Vivienda Joven
08/09/16	Diversas intervenciones durante la Inauguración del Centro de Innovación y Desarrollo de la Industria Mexicana de Coca-Cola
12/09/16	Diversas intervenciones durante la Inauguración de los Parques Ventika
12/09/16	Inauguración Parques Eólicos Ventika
19/09/16	El pacto mundial para una migración segura, regular y ordenada al que aspiramos, debe ver en el migrante a un aliado del desarrollo: EPN
19/09/16	Mensaje Presidente Enrique Peña Nieto en la Ceremonia de Entrega del Statesman Award de la Foreign Policy Association, en Nueva York, EUA
19/09/16	México seguirá participando activamente en Foros Multilaterales para concretar el Pacto Global para migración ordenada, segura y regular:EPN
19/09/16	Palabras de EPN, 71° Asamblea General ONU: Plenaria de la Reunión de Alto Nivel para atender grandes movimientos de migrantes y refugiados
19/09/16	Palabras Presidente EPN, Mesa Redonda 5: Pacto Global Migración Segura, Regular y Ordenada: Hacia el Logro de la Agenda 2030 para el DS
19/09/16	Reunión de Alto Nivel para Atender los Grandes Movimientos de Refugiados y Migrantes
20/09/16	Debate General de la Asamblea General de la ONU en su 71º Periodo de Sesiones
20/09/16	México llama a la Comunidad Internacional a ser solidaria con los refugiados y procurar su integración a las comunidades de destino: EPN
20/09/16	México refrenda compromiso con los principios democráticos, como único camino para la consecución de objetivos de desarrollo sostenible: EPN
20/09/16	Palabras del Presidente Enrique Peña Nieto, durante la Cumbre de Líderes sobre Refugiados, en el marco de la 71º Asamblea General de la ONU
20/09/16	Palabras del Presidente, licenciado Enrique Peña Nieto, durante su intervención en el Debate General de la Asamblea General de la ONU
21/09/16	Acelerar el crecimiento de las energías limpias, ayudará a conseguir los Objetivos de Desarrollo Sostenible y reforzará la estabilidad y la seguridad internacional.
23/09/16	Diversas intervenciones durante la Inauguración de la Planta de Tableros Tecnotabla de la Empresa PROTEAK UNO, S.A.B. de C.V.
23/09/16	México está comprometido en trabajar y hacer su aporte para mitigar y disminuir la emisión de gases efecto invernadero: Enrique Peña Nieto
23/09/16	Palabras Presidente Enrique Peña Nieto, durante la Inauguración de Planta de Tableros Tecnotabla de la Empresa PROTEAK UNO, S.A.B. de C.V.
23/09/16	Planta de Tableros Tecnotabla de la empresa PROTEAK UNO
06/10/16	Diversas intervenciones durante la Inauguración de la Fábrica de Nutrición Infantil "Nantli" de Nestlé
06/10/16	Diversas intervenciones durante la Reunión Anual de Industriales 2016, Comida Inaugural de la CONCAMIN de los Estados Unidos Mexicanos
24/10/16	Palabras Anthony Wayne Jerome Phillips-Spencer, Embajador República de Trinidad y Tobago, presentó sus Cartas Credenciales al Presidente EPN

Date	Title
04/11/16	Declaración Conjunta México – Suiza
04/11/16	Inversiones y comercio México - Suiza
07/11/16	Diversas intervenciones durante el 5º Foro Nacional Sumemos Causas, por la Seguridad: Ciudadanos + Policías
22/11/16	Diversas intervenciones durante la Designación del Bien Serial "Archipiélago de Revillagigedo" como Patrimonio Mundial de la Humanidad
05/12/16	Diversas intervenciones durante la Presentación del Decreto de Reserva de la Biosfera Caribe Mexicano
05/12/16	El Gobierno de la República da un paso histórico en materia de preservación y protección del medio ambiente: Enrique Peña Nieto
10/01/17	Así como la unidad es vital para enfrentar una emergencia natural, también es importante para resolver retos que ponen a prueba al país: EPN
10/01/17	Diversas intervenciones, Sesión Ordinaria del Consejo Nacional de Protección Civil y Entrega del Premio Nacional de Protección Civil 2016
10/01/17	México ha logrado avances significativos en Protección Civil
10/01/17	Palabras Presidente, EPN, Sesión Ordinaria del Consejo Nacional de Protección Civil y Entrega del Premio Nacional de Protección Civil 2016
11/01/17	Palabras del Presidente de los Estados Unidos Mexicanos, Enrique Peña Nieto, durante la 28 Reunión con Embajadores y Cónsules de México
13/01/17	Diversas intervenciones durante la Presentación del Estudio de la Política Turística de México
01/03/17	Central Eléctrica Pesquería
22/03/17	Diversas en la entrega de la Ampliación y Mejoramiento de la Red de Agua Potable de Acapulco; Inauguración Carretera Feliciano-Zihuatanejo
29/03/17	Convoca el Presidente Enrique Peña Nieto a los países mesoamericanos a atender las causas de migración
29/03/17	Palabras Presidente, licenciado Enrique Peña Nieto, durante la Sesión Plenaria de los Jefes de Estado y de Gobierno y Jefes de Delegación
29/03/17	Sesión Plenaria del Mecanismo de Diálogo y Concertación de Tuxtla
31/03/17	Consejo Nacional de la Agenda 2030 para el Desarrollo Sostenible
03/04/17	Declaración Conjunta entre el Gobierno de los Estados Unidos Mexicanos y el Gobierno del Reino de Dinamarca
03/04/17	Fortalecen México y Dinamarca relación; inaugurarán mañana, en Puerto de Lázaro Cárdenas, obras con capital danés por más de 7 mil 300 MDP
03/04/17	Mensaje a medios de comunicación del Primer Ministro de Dinamarca, Lars Lokke Rasmussen, en el marco de su Visita Oficial a México
03/04/17	Mensaje a medios que ofreció el Presidente Enrique Peña Nieto, por la Visita Oficial del Primer Ministro de Dinamarca, Lars Lokke Rasmussen
03/04/17	México y Dinamarca firman Declaración Conjunta
07/04/17	Diversas intervenciones durante la Inauguración del Parque Eólica de Coahuila, S.A. de C.V.
07/04/17	México se está volviendo más competitivo y más atractivo para la inversión: Enrique Peña Nieto
11/04/17	Mensajes que ofrecieron el licenciado Pedro Joaquín Coldwell…
24/04/17	Declaración Conjunta "Hacia una relación estratégica entre México y Polonia"
26/04/17	Diversas intervenciones durante la Instalación del Consejo Nacional de la Agenda 2030 para el Desarrollo Sostenible
26/04/17	Inaugura Presidente Enrique Peña Nieto la Primera Reunión del Foro de los países de América Latina y el Caribe sobre Desarrollo Sustentable
26/04/17	Instala el Presidente Enrique Peña Nieto el Consejo Nacional de la Agenda 2030 para el Desarrollo Sostenible
19/05/17	El Presidente Enrique Peña Nieto recibió al Gobernador del Estado de Washington, Estados Unidos de América
24/05/17	Acuerdos alcanzados en el Foro de Líderes
24/05/17	Diversas intervenciones durante la Ceremonia de Apertura de la Plataforma Global Reducción del Riesgo de Desastre 2017
24/05/17	Palabras del Presidente, Enrique Peña Nieto, en la Ceremonia de Apertura de la Plataforma Global de Reducción del Riesgo de Desastre 2017
24/05/17	Plataforma Global para la Reducción del Riesgo de Desastres 2017
25/05/17	Se reúnen el Presidente Enrique Peña Nieto y la Vicesecretaria General de la ONU, Amina J. Mohammed
07/06/17	México se compromete a proteger los ecosistemas marinos en peligro
09/06/17	El Presidente Enrique Peña Nieto recibió en el Palacio Nacional a la Canciller Federal de Alemania, Angela Merkel
10/06/17	Diversas intervenciones durante el evento: Alemania y México: Socios en el camino hacia la Industria 4.0 y la Formación Dual 4.0
20/06/17	México ve en la Globalización una fuerza positiva para el desarrollo y el progreso: Francisco Guzmán Ortiz

Date	Title
08/07/17	Cumbre de Líderes del G20
08/07/17	Mensaje del Presidente Enrique Peña Nieto al termino de su participacion en la cumbre de lideres del G20
17/07/17	Recibe el Presidente Enrique Peña Nieto al Mandatario Portugués, Marcelo Rebelo de Sousa
17/07/17	Visita de Estado del Presidente de la República Portuguesa, Marcelo Rebelo de Sousa
24/07/17	Diversas intervenciones durante la Entrega del Canal 27 de Enero y Canales Laterales
08/08/17	Diversas intervenciones durante el evento: El Mega Drenaje Pluvial de Campeche
08/08/17	El Presidente Enrique Peña Nieto inauguró en Campeche el Mega Drenaje Pluvial
15/08/17	Diversas intervenciones durante la Entrega del Libramiento Ferroviario de Durango
16/08/17	Diversas intervenciones durante Inicio de Pruebas de Operación de Central Ciclo Combinado Empalme I, en marco del 80 Aniversario de la CFE
16/08/17	La Comisión Federal de Electricidad brinda servicio al 98.6 por ciento de los mexicanos: Enrique Peña Nieto
25/08/17	El Presidente Enrique Peña Nieto se reúne con el Vicepresidente de la Confederación Suiza
02/09/17	#5toInforme: México con Responsabilidad Global
02/09/17	Hemos sentado las bases de un mejor país: Enrique Peña Nieto
02/09/17	Palabras del Presidente de los Estados Unidos Mexicanos, licenciado Enrique Peña Nieto, durante su Quinto Informe de Gobierno
09/10/17	Diversas intervenciones durante el 23 Congreso Internacional de Riego y Drenaje
09/10/17	El Riego Tecnificado junto con Estrategias para detonar el desarrollo del campo han permitido una nueva etapa de productividad: EPN
12/10/17	El Presidente Enrique Peña Nieto recibió al Primer Ministro de Canadá, Justin Trudeau
12/10/17	Mensaje a medios del Primer Ministro de Canadá, Justin Trudeau, en el marco de su Visita Oficial a México
12/10/17	Palabras del Primer Ministro de Canadá, Justin Trudeau, cena que ofreció el Presidente, Enrique Peña Nieto, en el marco de su Visita Oficial
12/10/17	Visita Oficial del Primer Ministro de Canadá, Justin Trudeau
18/10/17	El Presidente Enrique Peña Nieto se reúne con el Director Ejecutivo de Unilever, Paul Polman
18/10/17	Reunión con el Director Ejecutivo de Unilever, Paul Polman
24/10/17	Mensaje a medios de comunicación del Presidente, licenciado Enrique Peña Nieto, Visita Oficial del Primer Ministro de Santa Lucía
24/10/17	Mensaje a medios de comunicación del Primer Ministro de Santa Lucía, Allen Chastanet, durante su Visita Oficial
24/10/17	Reafirman México y Santa Lucía sus lazos de amistad y de cooperación
24/10/17	Visita Oficial del Primer Ministro de Santa Lucía, Allen Chastanet
25/10/17	IV Cumbre México-CARICOM
25/10/17	Palabras del Presidente de los Estados Unidos Mexicanos, licenciado Enrique Peña Nieto, durante la IV Cumbre México-CARICOM
25/10/17	Presenta el Presidente Peña Nieto ante países del Caribe la Estrategia de Gestión Integral de Riesgos de Desastres México-CARICOM
03/11/17	Vietnam y México: Uniendo al Pacífico
06/11/17	Diversas intervenciones durante la Ceremonia del Vertimiento Controlado del Casco del Ex Buque Destructor ARM "Comodoro Manuel Azueta" D-111
06/11/17	Exhorta el Presidente EPN a seguir preservando nuestra increíble biodiversidad, al mismo tiempo impulsamos la economía de familias mexicanas
09/11/17	Se reúne el Presidente Enrique Peña Nieto con el Presidente de la República Socialista de Vietnam, Trần Đại Quang
11/11/17	Concluye el Presidente Enrique Peña Nieto su participación en la XXV Reunión de Líderes de APEC
11/11/17	Concluye XXV Reunión de APEC
11/11/17	Mensaje a medios de comunicación del Presidente, EPN, en la 25ª Reunión de Líderes del Foro de Cooperación Económica Asia-Pacífico (APEC)
15/11/17	Declaración Conjunta México-República Oriental de Uruguay
21/11/17	Declaratoria Conjunta México-Eslovaquia
21/11/17	México y Eslovaquia fortalecen sus lazos de amistad y cooperación
21/11/17	Visita de Estado del Presidente de la República Eslovaca, Andrej Kiska
24/11/17	Diversas intervenciones durante la Firma de Decreto del Parque Nacional de Revillagigedo
07/12/17	Diversas intervenciones durante el evento: México Alimentaria Food Show 2017
12/12/17	Cumbre "One Planet" sobre Cambio Climático
12/12/17	Mantener con vida el Acuerdo de París sobre el clima
12/12/17	Participa el Presidente Peña Nieto en la Cumbre "One Planet" sobre Cambio Climático
13/12/17	Diversas intervenciones durante la 53 Reunión Ordinaria de la Conferencia Nacional de Gobernadores
26/12/17	Acciones y resultados en el Sector Ambiental en 2017

Date	Title
11/01/18	La grandeza de un país se mide por el bienestar de su pueblo y el valor de sus aportaciones al mundo: Enrique Peña Nieto
11/01/18	Palabras del Presidente de los Estados Unidos Mexicanos, Enrique Peña Nieto, durante la 29 Reunión de Embajadores y Cónsules de México
18/01/18	Declaración Conjunta México - República del Paraguay
20/02/18	Diversas intervenciones durante el inicio de Colecta Nacional Cruz Roja 2018
13/03/18	La firma del CPTPP es una parte fundamental del legado del Gobierno del Presidente Peña Nieto en materia comercial: Eduardo Sánchez H.
13/03/18	Mensajes que ofrecieron el Secretario de Economía, Ildefonso Guajardo Villarreal, y el Coordinador General de Comunicación Socia
12/04/18	Mensaje del Presidente, Enrique Peña Nieto, en el marco de la Visita Oficial de la Primera Ministra del Reino de Noruega, Erna Solberg
14/04/18	El Presidente Enrique Peña Nieto se reúne con el Vicepresidente de EUA, en el marco de la VIII Cumbre de las Américas
24/04/18	Declaración Conjunta México-Países Bajos
02/05/18	Diversas intervenciones durante la Cumbre de Financiamiento a la Vivienda INFONAVIT 2018
07/05/18	Palabras del Embajador de Santa Lucía, Anton Edmunds, durante la Presentación de sus Cartas Credenciales al Presidente, Enrique Peña Nieto
07/05/18	Palabras Embajador de Nueva Zelandia, Mark Sinclair, durante la Presentación de sus Cartas Credenciales al Presidente, Enrique Peña Nieto
08/05/18	Diversas intervenciones en la Clausura de la 34 Asamblea CNA y Presentación Estrategia Atención Integral para Jornaleros Agrícolas Migrantes
08/05/18	México refrenda su compromiso con el desarrollo sostenible de América Latina y el Caribe
25/05/18	Intervenciones Sesión del Consejo Nacional de Protección Civil. Premio Nacional Protección Civil 2017 e Inicio de Temporada de Lluvias 2018
28/05/18	Diversas intervenciones durante el Encuentro con los Usuarios de Riego
05/06/18	Diversas intervenciones durante la Promulgación de la Ley General de Desarrollo Forestal Sustentable y Firma de Decretos de Reserva de Agua
05/06/18	Palabras Presidente, Enrique Peña Nieto, Promulgación Ley General de Desarrollo Forestal Sustentable y Firma de Decretos de Reserva de Agua
05/06/18	Promulga el Presidente Enrique Peña Nieto la Ley General de Desarrollo Forestal Sustentable
05/06/18	Promulgación de Ley General de Desarrollo Sustentable y firma de Decretos de Reserva de Agua
19/06/18	Avances en la actualización de la CFE
19/06/18	La CFE ha dado grandes pasos en modernización de infraestructura y en el uso de fuentes de energía más eficientes, limpias y económicas: ESH
19/06/18	Mensajes que ofrecieron el doctor Jaime Hernández Martínez, Director General de la Comisión Federal de Electricidad
16/08/18	Palabras del Presidente de los Estados Unidos Mexicanos
03/09/18	Hoy somos un país mejor del que éramos hace seis años: Enrique Peña Nieto
03/09/18	Palabras del Presidente de los Estados Unidos Mexicanos, licenciado Enrique Peña Nieto, con motivo de su Sexto Informe de Gobierno
07/09/18	Diversas intervenciones durante la Entrega del Premio Nacional de Exportación y Clausura del XXV Congreso del Comercio Exterior Mexicano
24/09/18	Panel de Alto Nivel para Construir una Economía Oceánica Sostenible
25/09/18	Participa el Presidente Peña Nieto en el Panel de Alto Nivel para Construir una Economía Oceánica Sostenible
25/09/18	Participación del Presidente, licenciado Enrique Peña Nieto, durante la 73° Asamblea General de la Organización de las Naciones Unidas
26/09/18	73a Asamblea General de las Naciones Unidas
26/09/18	Concluye el Presidente Enrique Peña Nieto sus actividades en la ONU
09/10/18	Clausura 13ª Conferencia de Ministros de Defensa de las Américas
09/10/18	Sumando esfuerzos, voluntades y recursos, consolidaremos una zona de paz, seguridad y desarrollo sostenible en el hemisferio americano: EPN
10/10/18	Palabras del Presidente, Enrique Peña Nieto, durante la Clausura de la 13ª Conferencia de Ministros de Defensa de las Américas
20/10/18	Diversas Intervenciones
25/10/18	El Presidente Enrique Peña Nieto recibió al Ministro de Europa y Asuntos Exteriores de Francia, Jean-Yves Le Drian
09/11/18	Diversas intervenciones, durante el Foro de Alcaldes por la Nueva Agenda Urbana, ONU-HABITAT-INFONAVIT
